# Generalizable biomarker prediction from cancer pathology slides with self-supervised deep learning: A retrospective multi-centric study

**DOI:** 10.1016/j.xcrm.2023.100980

**Published:** 2023-03-22

**Authors:** Jan Moritz Niehues, Philip Quirke, Nicholas P. West, Heike I. Grabsch, Marko van Treeck, Yoni Schirris, Gregory P. Veldhuizen, Gordon G.A. Hutchins, Susan D. Richman, Sebastian Foersch, Titus J. Brinker, Junya Fukuoka, Andrey Bychkov, Wataru Uegami, Daniel Truhn, Hermann Brenner, Alexander Brobeil, Michael Hoffmeister, Jakob Nikolas Kather

**Affiliations:** 1 Else Kroener Fresenius Center for Digital Health, Technical University Dresden, 01307 Dresden, Germany; 2 Department of Medicine III, University Hospital RWTH Aachen, 52074 Aachen, Germany; 3 Pathology & Data Analytics, Leeds Institute of Medical Research at St James’s, University of Leeds, Leeds LS9 7TF, UK; 4 Department of Pathology, GROW School for Oncology and Reproduction, Maastricht University Medical Center+, 6229 HX Maastricht, the Netherlands; 5 Netherlands Cancer Institute, 1066 CX Amsterdam, the Netherlands; 6 University of Amsterdam, 1012 WP Amsterdam, the Netherlands; 7 Institute of Pathology, University Medical Center Mainz, 55131 Mainz, Germany; 8 Digital Biomarkers for Oncology Group, German Cancer Research Center (DKFZ), 69120 Heidelberg, Germany; 9 Department of Pathology Informatics, Nagasaki University Graduate School of Biomedical Sciences, Nagasaki 852-8523, Japan; 10 Department of Pathology, Kameda Medical Center, Kamogawa 296-8602, Chiba, Japan; 11 Department of Diagnostic and Interventional Radiology, University Hospital RWTH Aachen, 52074 Aachen, Germany; 12 Division of Clinical Epidemiology and Aging Research, German Cancer Research Center (DKFZ), 69120 Heidelberg, Germany; 13 Division of Preventive Oncology, German Cancer Research Center (DKFZ) and National Center for Tumor Diseases (NCT), 69120 Heidelberg, Germany; 14 German Cancer Consortium (DKTK), German Cancer Research Center (DKFZ), 69120 Heidelberg, Germany; 15 Institute of Pathology, University Hospital Heidelberg, 69120 Heidelberg, Germany; 16 Tissue Bank, National Center for Tumor Diseases (NCT), University Hospital Heidelberg, 69120 Heidelberg, Germany; 17 Department of Medicine I, University Hospital Dresden, 01307 Dresden, Germany; 18 Medical Oncology, National Center for Tumor Diseases (NCT), University Hospital Heidelberg, 69120 Heidelberg, Germany

**Keywords:** artificial intelligence, biomarker, colorectal cancer, computational pathology, oncogenic mutation, multi-input models, attention-based multiple-instance learning, self-supervised learning, attention heatmaps

## Abstract

Deep learning (DL) can predict microsatellite instability (MSI) from routine histopathology slides of colorectal cancer (CRC). However, it is unclear whether DL can also predict other biomarkers with high performance and whether DL predictions generalize to external patient populations. Here, we acquire CRC tissue samples from two large multi-centric studies. We systematically compare six different state-of-the-art DL architectures to predict biomarkers from pathology slides, including MSI and mutations in *BRAF*, *KRAS*, *NRAS*, and *PIK3CA*. Using a large external validation cohort to provide a realistic evaluation setting, we show that models using self-supervised, attention-based multiple-instance learning consistently outperform previous approaches while offering explainable visualizations of the indicative regions and morphologies. While the prediction of MSI and *BRAF* mutations reaches a clinical-grade performance, mutation prediction of *PIK3CA*, *KRAS*, and *NRAS* was clinically insufficient.

## Highlights

• Deep learning can predict MSI and *BRAF* status from routine pathology slides
• Predictions for *KRAS*, *NRAS*, and *PIK3CA* mutations are below clinical-grade performance
• Multi-input models generalize better for BRAF biomarker prediction

## Graphical abstract

**Figure:**
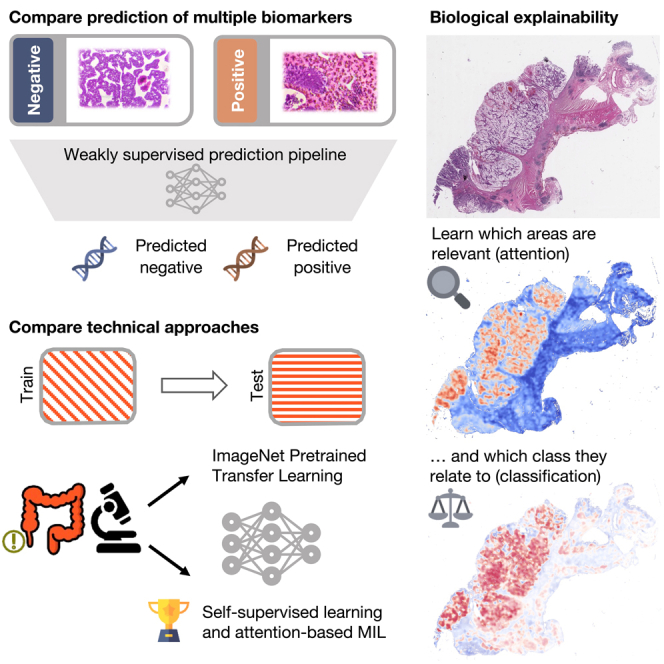

## Teaser

Niehues et al. evaluate deep-learning-based prediction for MSI, *BRAF*, *KRAS*, *NRAS*, and *PIK3CA* biomarker status in colorectal cancer from histopathology slides. They evaluate the performances of trained models in a realistic setting on a large independent patient cohort and find that attention-based multiple-instance learning outperforms all other approaches.

## Introduction

Digitized histopathological slides with hematoxylin and eosin (H&E) staining offer a wealth of information that can be quantified and made usable by artificial intelligence (AI), in particular by deep learning (DL) neural networks.^1^ DL networks have been developed to predict clinically relevant biomarkers directly from H&E-stained tumor tissue sections.^2^^,^^3^^,^^4^^,^^5^ The application of DL for such complex tasks represents a major part of “computational pathology.”^3^^,^^4^^,^^6^ In colorectal cancer (CRC), DL-based predictability of biomarkers from H&E-stained tissue sections has been reported for microsatellite instability (MSI)^7^^,^^8^^,^^9^^,^^10^^,^^11^^,^^12^^,^^13^^,^^14^ and, in smaller studies, for mutations in *BRAF*,^10^^,^^13^ *TP53*, *KRAS*, *SMAD4, PIK3CA*, and other genes.^4^^,^^15^^,^^16^ Prediction of MSI or mismatch repair deficiency (dMMR) in CRC is one of the most widely studied tasks^17^ due to its high clinical relevance: first, the MSI status may point to hereditary causes of CRC.^18^ Second, MSI is the strongest predictor of response to cancer immunotherapy.^19^ Third, MSI has an important role in the management of patients with CRC, for example in the decision whether to prescribe adjuvant chemotherapy.^20^

Building on evidence provided in multiple studies,^7^^,^^9^^,^^14^^,^^17^^,^^21^^,^^22^ the first DL algorithm for MSI prediction has received regulatory approval in Europe in 2022 (“MSIntuit CRC” by Owkin, France/USA). However, various questions remain open, which is even more relevant now that this method can be used in routine diagnostics. The most important issue of existing MSI detection algorithms is their generalizability.^23^ Usually, a pronounced performance drop is observed when deploying the trained models on external patient cohorts.^21^ Validation on external cohorts is crucial for testing the translation of models’ prediction performance and hence generalization to independent datasets. The second issue is explainability, i.e., identifying which tissue patterns are associated with which genetic alterations. The third issue is the scope of the methods, i.e., their application to other biomarkers beyond MSI. Many genetic alterations are related to morphological features in tumor tissue. This is known for MSI^24^ and *BRAF* mutations^25^ in CRC and several mutations in other tumor types.^26^^,^^27^ However, few studies have investigated alterations beyond MSI in CRC in large patient cohorts. While recent studies investigating the DL-based prediction of MSI status included thousands of patients,^17^ studies investigating other biomarkers such as *BRAF*, *KRAS*, *NRAS*, and *PIK3CA* mutations are often limited to smaller cohorts with suboptimal data quality.^28^

From a technical point of view, the most widely used method for biomarker prediction in computational pathology is to train DL networks on image tiles obtained from histological whole-slide images (WSI).^4^^,^^29^ Mutation labels, however, only exist for the entire WSI, and it is unclear which regions on the WSI express morphologies that reflect underlying mutations. Therefore, tile predictions must be aggregated to slide predictions. A common approach is to apply transfer learning to models pre-trained on ImageNet and to use mean pooling for tile-to-slide aggregation.^7^^,^^29^^,^^30^^,^^31^ This method, the ImageNet pre-trained (INPT) approach, was first applied in histopathology by Coudray et al. in 2018.^30^ Recent proof-of-concept studies have suggested that the attention-based multiple-instance learning (attMIL)^32^ approach is superior to the INPT approach.^12^ The image feature extractor (encoder) in attMIL can be pre-trained via self-supervised learning (SSL). Schirris et al. used SSL-attMIL in a pilot study on a public dataset with 360 patients.^12^ On this relatively small dataset, they reported a performance gain compared with the INPT approach. However, this performance gain has not been validated in larger cohorts. Similarly, other works have applied the attMIL approach with and without SSL to predict biomarkers but have only provided external validation in small datasets, if at all.^5^^,^^33^^,^^34^ In summary, previous evidence suggests that both SSL and attMIL are useful components in weakly supervised computational pathology pipelines, but this has not been systematically tested in a clinically relevant task with large-scale external validation. Such a lack of large-scale validation is a risk for the ultimate generalizability of any biomarker.^23^^,^^35^

In this light, we aimed to fill two knowledge gaps by answering two questions: first, do attMIL and SSL really provide a performance gain compared with the INPT approach? Second, is MSI the only predictable biomarker in CRC, or is the mutational status of BRAF, KRAS, NRAS, and PIK3CA similarly predictable?

To this end, we implemented the INPT approach as a baseline and trained models for the prediction of multiple biomarkers in CRC. We tested the generalization on a test dataset and saw a performance drop, as expected. Subsequently, we implemented attMIL and applied it using two different SSL-trained feature extractors. We showed that one encoder outperformed the other by a large margin. The better encoder generalized well to the second dataset and consistently outperformed all other tested models. Finally, we extended attMIL by including clinical patient data and show that there was no synergy for the performance on the training dataset, although performance on the test dataset was increased.

## Results

### attMIL outperforms the INPT approach for biomarker prediction

**Figure 1:**
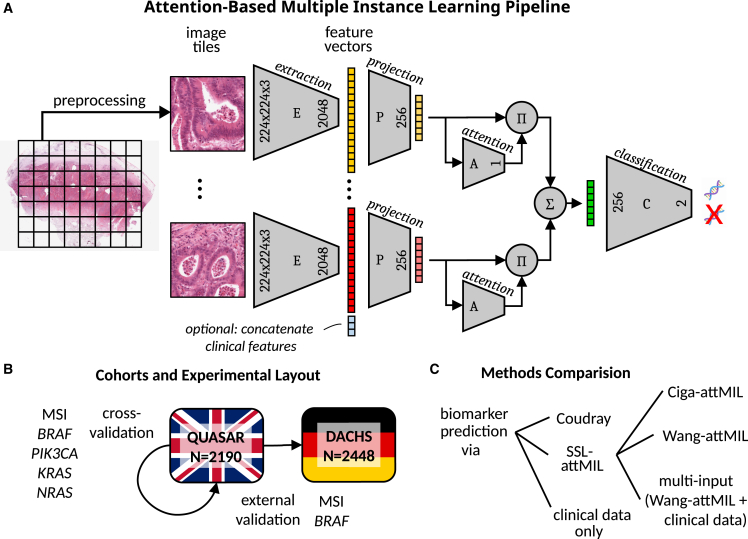
Schematic workflow of this study (A) Schematic summary of attMIL and the multi-input DL architecture: a WSI is tessellated into smaller tiles, that are subsequently pre-processed and passed through the encoder to give image feature vectors. In the multi-input case, each image feature vector is concatenated by a vector representing the patient’s clinical data. The set of image feature vectors per WSI is then used as input to the attMIL model. In a first embedding block, the attMIL model reduces the dimension of each tile’s initial feature vector to 256 (from 2,048 [+4 if clinical data are used in the input] when using the Wang encoder). Then, the attention score per tile is calculated. Using the attention score, the attention-weighted sum over all embedded feature vectors can be evaluated to give a 256-dimensional vector representing the entire WSI (green). Finally, this vector is passed through a classification block to obtain a biomarker prediction for the input WSI. (B) Targets and cohorts used in internal and external validation. For internal validation, we tested for MSI, *BRAF*, *PIK3CA*, *KRAS*, and *NRAS* status. Externally only for MSI and *BRAF* status. (C) List of all six DL approaches that were compared in this study. E, encoder network; P, embedding block that embeds feature vectors into a lower dimensional space; A, attention layers; Π, attention weighting; Σ, sum; C, classification block.

**Figure 2:**
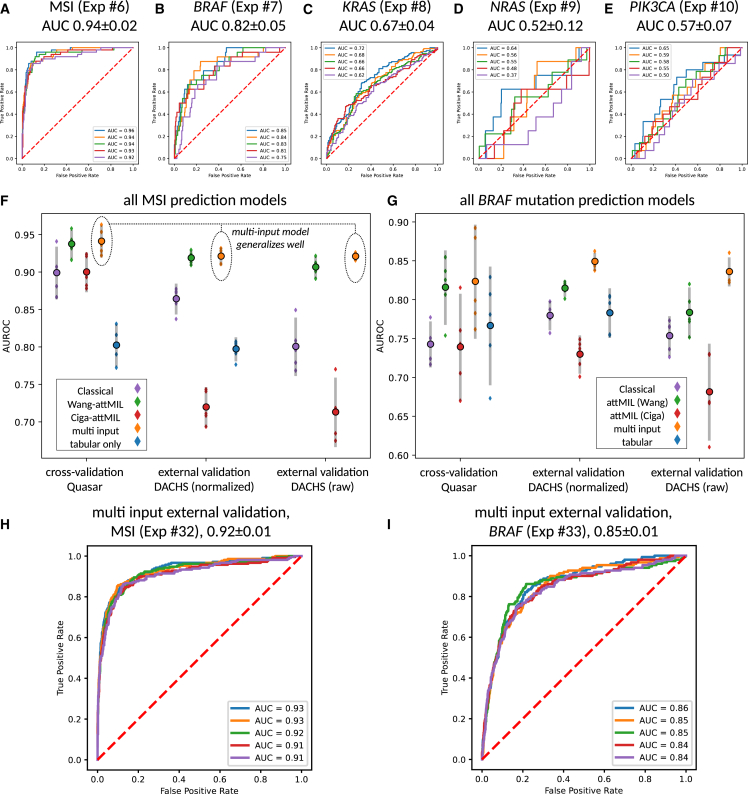
Biomarker prediction performance of deep-learning models (A–E) Cross-validated AUROCs for all biomarkers obtained using the Wang-attMIL model. (F and G) Internal cross-validated performance of all models on QUASAR and external validation on DACHS (with and without Macenko color normalization). The bar charts show the distribution of five technical replicates and error bars indicate 95% confidence intervalls. In internal cross-validation, replicates are separate cross-validation runs. In external validation, replicates are deployments of the individual cross-validation models. Central markers give the average AUROC score in each setup. (H and I) The error bars indicate 95% confidence intervals AUROCs obtained by models trained in each of the five folds for MSI and *BRAF* status prediction, applied to the external validation set QUASAR.

First, we investigated the predictability of MSI, *BRAF*, *KRAS*, *NRAS*, and *PIK3CA* directly from H&E histopathology images in the QUASAR cohort (Tables 1 and 2). We compared the INPT approach with SSL-attMIL using the SSL encoders by Ciga or Wang (Figures 1A–1C). We found that the best performances were obtained using image-only Wang-attMIL. For prediction of MSI, *BRAF*, *KRAS*, *NRAS*, and *PIK3CA*, areas under the receiver operating characteristic curve (AUROCs) of 0.94 ± 0.02, 0.82 ± 0.05, 0.67 ± 0.04, 0.52 ± 0.12, and 0.57 ± 0.07 were obtained, respectively (Figures 2A–2E). Previous studies have discussed that AUROCs of close to 0.9 with good generalization have a high discriminative power, which may be clinically relevant.^9^^,^^29^^,^^36^^,^^37^ In this sense, only MSI and *BRAF* mutation prediction reached a potentially clinically relevant level, but the prediction of the other investigated biomarkers did not.

**Table 1:** All experimental results

Exp.	Target	Train	Test	Algorithm	Feats.	AUROC	AUPRCpos	AUPRCneg	Norm in test
1	MSI	QUASAR	QUASAR	INPT	n.a.	0.90 ± 0.04	0.63 ± 0.09	0.98 ± 0.01	Macenko
2	*BRAF*	QUASAR	QUASAR	INPT	n.a.	0.74 ± 0.03	0.25 ± 0.06	0.97 ± 0.01	Macenko
3	*KRAS*	QUASAR	QUASAR	INPT	n.a.	0.63 ± 0.06	0.52 ± 0.07	0.72 ± 0.05	Macenko
4	*NRAS*	QUASAR	QUASAR	INPT	n.a.	0.50 ± 0.08	0.04 ± 0.01	0.97 ± 0.01	Macenko
5	*PIK3CA*	QUASAR	QUASAR	INPT	n.a.	0.54 ± 0.04	0.07 ± 0.02	0.95 ± 0.01	Macenko
6	MSI	QUASAR	QUASAR	attMIL	Wang	0.94 ± 0.02	0.76 ± 0.04	0.99 ± 0.01	Macenko
7	*BRAF*	QUASAR	QUASAR	attMIL	Wang	0.82 ± 0.05	0.36 ± 0.13	0.98 ± 0.01	Macenko
8	*KRAS*	QUASAR	QUASAR	attMIL	Wang	0.67 ± 0.04	0.57 ± 0.05	0.74 ± 0.05	Macenko
9	*NRAS*	QUASAR	QUASAR	attMIL	Wang	0.52 ± 0.12	0.05 ± 0.04	0.97 ± 0.01	Macenko
10	*PIK3CA*	QUASAR	QUASAR	attMIL	Wang	0.57 ± 0.07	0.07 ± 0.02	0.96 ± 0.01	Macenko
11	MSI	QUASAR	QUASAR	attMIL	Ciga	0.90 ± 0.03	0.64 ± 0.09	0.98 ± 0.01	Macenko
12	*BRAF*	QUASAR	QUASAR	attMIL	Ciga	0.74 ± 0.07	0.24 ± 0.08	0.96 ± 0.01	Macenko
13	*KRAS*	QUASAR	QUASAR	attMIL	Ciga	0.59 ± 0.03	0.48 ± 0.05	0.69 ± 0.03	Macenko
14	*NRAS*	QUASAR	QUASAR	attMIL	Ciga	0.58 ± 0.15	0.04 ± 0.02	0.98 ± 0.01	Macenko
15	*PIK3CA*	QUASAR	QUASAR	attMIL	Ciga	0.57 ± 0.15	0.12 ± 0.08	0.96 ± 0.02	Macenko
16	MSI	QUASAR	QUASAR	multi-input	Wang	0.94 ± 0.02	0.77 ± 0.07	0.99 ± 0.01	Macenko
17	*BRAF*	QUASAR	QUASAR	multi-input	Wang	0.82 ± 0.07	0.43 ± 0.11	0.98 ± 0.01	Macenko
18	*KRAS*	QUASAR	QUASAR	multi-input	Wang	0.66 ± 0.04	0.57 ± 0.03	0.74 ± 0.05	Macenko
19	*NRAS*	QUASAR	QUASAR	multi-input	Wang	0.49 ± 0.18	0.08 ± 0.07	0.97 ± 0.02	Macenko
20	*PIK3CA*	QUASAR	QUASAR	multi-input	Wang	0.52 ± 0.17	0.09 ± 0.10	0.95 ± 0.03	Macenko
21	MSI	QUASAR	QUASAR	clinical data only	n.a.	0.80 ± 0.03	0.37 ± 0.05	0.96 ± 0.01	n.a.
22	*BRAF*	QUASAR	QUASAR	clinical data only	n.a.	0.77 ± 0.08	0.24 ± 0.07	0.96 ± 0.02	n.a.
23	*KRAS*	QUASAR	QUASAR	clinical data only	n.a.	0.50 ± 0.06	0.41 ± 0.05	0.62 ± 0.04	n.a.
24	*NRAS*	QUASAR	QUASAR	clinical data only	n.a.	0.54 ± 0.13	0.06 ± 0.07	0.98 ± 0.01	n.a.
25	*PIK3CA*	QUASAR	QUASAR	clinical data only	n.a.	0.59 ± 0.06	0.08 ± 0.02	0.97 ± 0.01	n.a.
26	MSI	QUASAR	DACHS	INPT	n.a.	0.86 ± 0.02	0.54 ± 0.04	0.98 ± 0.01	Macenko
27	*BRAF*	QUASAR	DACHS	INPT	n.a.	0.78 ± 0.02	0.22 ± 0.01	0.98 ± 0.00	Macenko
28	MSI	QUASAR	DACHS	attMIL	Wang	0.92 ± 0.01	0.68 ± 0.03	0.99 ± 0.00	Macenko
29	*BRAF*	QUASAR	DACHS	attMIL	Wang	0.81 ± 0.01	0.27 ± 0.02	0.98 ± 0.00	Macenko
30	MSI	QUASAR	DACHS	attMIL	Ciga	0.72 ± 0.03	0.32 ± 0.05	0.95 ± 0.00	Macenko
31	*BRAF*	QUASAR	DACHS	attMIL	Ciga	0.73 ± 0.02	0.18 ± 0.01	0.97 ± 0.01	Macenko
32	MSI	QUASAR	DACHS	multi-input	Wang	0.92 ± 0.01	0.72 ± 0.03	0.99 ± 0.00	Macenko
33	*BRAF*	QUASAR	DACHS	multi-input	Wang	0.85 ± 0.01	0.35 ± 0.02	0.98 ± 0.00	Macenko
34	MSI	QUASAR	DACHS	clinical data only	n.a.	0.80 ± 0.02	0.28 ± 0.04	0.97 ± 0.00	n.a.
35	*BRAF*	QUASAR	DACHS	clinical data only	n.a.	0.78 ± 0.03	0.22 ± 0.04	0.97 ± 0.01	n.a.
36	MSI	QUASAR	DACHS	INPT	n.a.	0.80 ± 0.04	0.33 ± 0.08	0.97 ± 0.01	none
37	*BRAF*	QUASAR	DACHS	INPT	n.a.	0.75 ± 0.03	0.18 ± 0.03	0.97 ± 0.00	none
38	MSI	QUASAR	DACHS	attMIL	Wang	0.91 ± 0.01	0.67 ± 0.03	0.98 ± 0.00	none
39	*BRAF*	QUASAR	DACHS	attMIL	Wang	0.78 ± 0.03	0.26 ± 0.04	0.97 ± 0.00	none
40	MSI	QUASAR	DACHS	attMIL	Ciga	0.71 ± 0.05	0.25 ± 0.09	0.95 ± 0.01	none
41	*BRAF*	QUASAR	DACHS	attMIL	Ciga	0.68 ± 0.06	0.15 ± 0.04	0.96 ± 0.01	none
42	MSI	QUASAR	DACHS	multi-input	Wang	0.92 ± 0.01	0.72 ± 0.02	0.99 ± 0.00	none
43	*BRAF*	QUASAR	DACHS	multi-input	Wang	0.84 ± 0.02	0.35 ± 0.04	0.98 ± 0.00	none
44	*BRAF*	QUASAR/MSI subgroup	QUASAR/MSI subgroup	attMIL	Wang	0.73 ± 0.06	0.63 ± 0.09	0.84 ± 0.03	Macenko
45	*BRAF*	QUASAR/MSS subgroup	QUASAR/MSS subgroup	attMIL	Wang	0.66 ± 0.10	0.12 ± 0.08	0.98 ± 0.01	Macenko
46	MSI	QUASAR/*BRAF*^wt^	QUASAR/*BRAF*^wt^	attMIL	Wang	0.89 ± 0.06	0.57 ± 0.11	0.98 ± 0.02	Macenko
47	MSI	QUASAR/*BRAF*^mut^	QUASAR/*BRAF*^mut^	attMIL	Wang	0.78 ± 0.15	0.80 ± 0.15	0.79 ± 0.15	Macenko

If the test set is the same as the training set, then five-fold cross-validation on the patient level was used. AUROCs are given as mean ± 95% confidence intervals for the five-fold AUC scores. Precision-recall curves for experiments 32 and 33 can be found in Figure S3. Domain shift plots for experiments 6, 7, 28, and 29 can be found in Figure S4 and for experiments 16, 17, 32, and 33 in Figure S5, respectively. Ex., experiment number; Feats., features; Norm, color normalization of the test set (training set was always color normalized).

**Table 2:** Clinico-pathological features of both cohorts

Patient/tumor characteristics	QUASAR	DACHS
Origin	United Kingdom	Germany
Number of patients	2,190	2,448
WSI format	SVS	SVS
MSI/dMMR ground truth	IHC 4-plex or IHC 2-plex	PCR 3-plex
MSI/dMMR, n (%)	246 (11%)	210 (9%)
MSS/pMMR, n (%)	1,529 (70%)	1,836 (75%)
Mean age at diagnosis (standard deviation)	62.20 (±9.60)	68.46 (±10.82)
Colon cancer, n (%)	1,474 (67%)	1,488 (61%)
Rectal cancer, n (%)	526 (24%)	960 (39%)
Organ unknown, n (%)	190 (9%)	0 (0%)
Female, n (%)	848 (39%)	1,012 (41%)
Male, n (%)	1,334 (61%)	1,436 (59%)
Gender unknown (%)	8 (0%)	0 (0%)
UICC stage I, n (%)	1 (0%)	485 (20%)
UICC stage II, n (%)	1,988 (91%)	801 (33%)
UICC stage III, n (%)	192 (9%)	822 (34%)
UICC stage IV, n (%)	0 (0%)	337 (14%)
UICC stage unknown (%)	9 (0%)	3 (0%)
*BRAF* mutation, n (%)	120 (5%)	151 (6%)
*BRAF* wild type, n (%)	1,358 (62%)	1,930 (79%)
*BRAF* status unknown (%)	712 (33%)	367 (15%)
*KRAS* mutation, n (%)	555 (25%)	667 (27%)
*KRAS* wild type, n (%)	882 (40%)	1,397 (57%)
*KRAS* status unknown (%)	753 (35%)	374 (15%)
*NRAS* mutation, n (%)	41 (2%)	n.a.
*NRAS* wild type, n (%)	1,430 (65%)	n.a.
*NRAS* status unknown (%)	719 (33%)	n.a.
*PIK3CA* mutation, n (%)	72 (3%)	n.a.
*PIK3CA* wild type, n (%)	1,343 (61%)	n.a.
*PIK3CA* status unknown (%)	775 (36%)	n.a.
Right-sided tumor, n (%)	754 (34%)	819 (33%)
Left-sided tumor, n (%)	1,158 (53%)	1,607 (66%)
Sidedness unknown, n (%)	150 (13%)	22 (1%)
Etiology	not specified	any

Details on missing image and/or biomarker data for patients in the QUASAR and DACHS cohort can be found in Figures S1 and S2, respectively.

**Figure 3:**
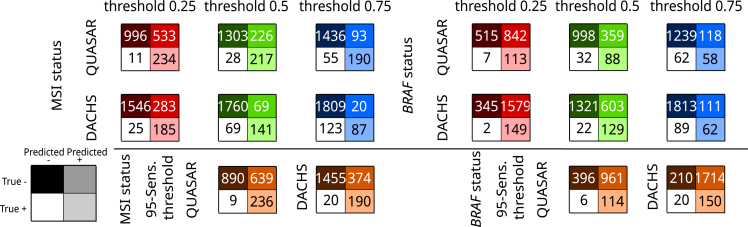
Test statistics for a potential screening tool using the Wang-attMIL image-only models Test performances at thresholds of 0.25, 0.5, and 0.75 (top) and at a threshold that yielded 95% in-domain sensitivity (95-Sens. threshold) averaged across the five models per biomarker. In-domain performances are measured by the summed model predictions over respective test sets. External performances on DACHS are obtained by averaging scores for biomarker prediction over all five Wang-attMIL models per biomarker. Clinical statistics for correctly classified and misclassified patients in QUASAR and DACHS at a threshold value of 0.5 are given in Tables S7 and S8.

Because using the AUROC as the sole metric is suboptimal,^38^ we evaluated the model performance of the image-only Wang-attMIL model in Quasar at pre-defined threshold values (Figure 3). For MSI prediction, the 95% in-domain sensitivity threshold of value 0.21 yielded 236 true positive, 639 false positive, 9 false negative, and 890 true negative predictions across the five internal datasets. This globally corresponds to a sensitivity of 96.3%, a specificity of 58.2%, a positive predictive value (PPV) of 27%, and a negative predictive value (NPV) of 99%. At a threshold value of 0.5, *BRAF* status was globally predicted with a sensitivity of 73.3%, a specificity of 73.5%, a PPV of 19.7%, and an NPV of 96.9% across the five internal test sets. For *BRAF* status prediction notably, the requirement of 95% in-domain sensitivity comes at a high cost in specificity.

The INPT approach performed slightly but statistically significantly worse (p < 0.05, for MSI and *BRAF*) than the image-only Wang-attMIL model, achieving AUROCs of 0.90 ± 0.04, 0.74 ± 0.03, 0.63 ± 0.06, 0.50 ± 0.08, and 0.54 ± 0.04 for MSI, *BRAF*, *KRAS*, *NRAS*, and *PIK3CA*, respectively. The Ciga-attMIL models yielded similar results as the INPT approach (AUROCs: MSI 0.90 ± 0.03, *BRAF* 0.74 ± 0.07, *KRAS* 0.59 ± 0.03, *NRAS* 0.58 ± 0.15, *PIK3CA* 0.57 ± 0.15), where MSI- and *BRAF*-status prediction performances are compatible between the two approaches.

Together, these data show that the DL methods presented in this article have the potential to reach clinical-grade performance for the prediction of MSI, and near-clinical-grade performance for the prediction of *BRAF* but that they do not reach a high performance for *KRAS*, *NRAS*, and *PIK3CA*, despite using the best-performing image-only Wang-attMIL models in a large patient cohort.

### There is no direct synergy between clinical data and image data in biomarker prediction

Further, we investigated whether or not adding baseline clinical data (gender, age, tumor location) as additional inputs improves the internal prediction performance of the best model. Wang-attMIL with clinical data (multi-input model) achieved the following AUROCs: MSI 0.94 ± 0.02, *BRAF* 0.82 ± 0.07 (Figures 2F and 2G), *KRAS* 0.66 ± 0.04, *NRAS* 0.49 ± 0.18, and *PIK3CA* 0.52 ± 0.17 (Table 1), yielding statistical compatibility with the image-only Wang-attMIL model for MSI and *BRAF* prediction. The solely clinical-data-based model achieved good prediction results as well (AUROCs: MSI 0.80 ± 0.03, *BRAF* 0.77 ± 0.08, Figures 2F and 2G; *KRAS* 0.50 ± 0.06, *NRAS* 0.54 ± 0.13, *PIK3CA* 0.59 ± 0.06, Table 1). In particular, the solely clinical-data-based results for *BRAF* mutation prediction were close to those obtained with the image-only Wang-attMIL or the multi-input model and statistically compatible with all other DL approaches. This indicates that the visual features on H&E∗stained tissue sections that are predictive of *BRAF* status are by themselves only slightly superior to the clinical variables. The same applies to the prediction of *NRAS* and *PIK3CA* mutation status. For *KRAS* and MSI status prediction, the image-based models outperformed the solely clinical-data-based model. This indicates better predictability of biomarker status from image features than from clinical variables for these two biomarkers.

### Image-only and multi-input attMIL generalizes better than the state of the art

Next, we assessed the generalizability of QUASAR-trained models on the DACHS cohort (Table 2; Figures S1 and S2). One set of tiles was color normalized using the Macenko method, while another set contained the same tiles without any color normalization. Here, we restricted the analysis to MSI and *BRAF* biomarker prediction, as other biomarkers had already been shown to perform poorly during internal validation.

The image-only Wang-attMIL models and the multi-input models yielded a high performance for the prediction of MSI and *BRAF* status (Figures 2F and 2G). For MSI and *BRAF* prediction on color-normalized tiles in the external validation cohort, AUROCs of 0.92 ± 0.01 and 0.81 ± 0.01 and 0.92 ± 0.01 and 0.85 ± 0.01 were obtained by image-only Wang-attMIL and multi-input, respectively (Figures 2F–2I). For *BRAF* mutation prediction, this shows a better generalization of the multi-input compared with the image-only Wang-attMIL models. These high AUROCs correspond to high areas under the precision-recall curve (AUPRCs) (Table 1; Figure S3), pointing to potential clinical applicability. For MSI prediction in DACHS with the 95% in-domain sensitivity threshold value of 0.21, the averaged models’ scores achieved a sensitivity of 90.5%, a specificity of 79.6%, a PPV of 33.7%, and an NPV of 98.6%. At a threshold value of 0.5, *BRAF* status was predicted with a sensitivity of 73.3%, a specificity of 73.5%, a PPV of 19.7%, and an NPV of 96.9% (Figure 3). Clinical statistics for correctly classified and misclassified patients in QUASAR and DACHS at a threshold value of 0.5 are given in Tables S1 and S2. The models had difficulties in correctly predicting MSI-positive patients with rectal cancer in the DACHS cohort. In the case of rectal carcinomas, the odds ratio for the correct classification of an MSI-positive patient in QUASAR compared with DACHS was 11.7, suggesting that more data from patients with rectal carcinoma are required in future datasets.

Notably, when using the Wang encoder, the performance in the validation cohort was not dependent on the presence of color normalization—we observed an equivalent performance on non-color-normalized tiles (image-only Wang-attMIL: MSI 0.91 ± 0.01, *BRAF* 0.78 ± 0.03; multi-input: MSI 0.92 ± 0.01, 0.84 ± 0.02). Here the multi-input outperformed the image-only Wang-attMIL models for both MSI and *BRAF* status prediction. This provides further evidence that (1) the image-only Wang-attMIL models generalize very well and do not suffer from domain shifts and that (2) addition of clinical data can improve generalization even further. Thus, combining morphological features with the patient’s age, gender, and tumor location can improve performance.

In contrast, the INPT models trained on QUASAR showed a marked performance drop on color-normalized DACHS images (AUROCs: MSI 0.86 ± 0.02, *BRAF* 0.78 ± 0.02) and further dropped in performance for the non-normalized images (AUROCs: MSI 0.80 ± 0.04, *BRAF* 0.75 ± 0.03). This shows that the INPT approach is less stable and generalizes less well than the image-only Wang-attMIL or multi-input models. The robustness of the Wang-attMIL approach seemed to be due to the particular encoder since the Ciga-attMIL model generalized poorly (AUROCs color normalized: MSI 0.72 ± 0.03, *BRAF* 0.73 ± 0.02; AUROCs non-normalized: MSI 0.71 ± 0.05, *BRAF* 0.68 ± 0.06; Table 1). Results for the analysis of variances (ANOVA) for AUROCs obtained with trained models in internal validation and in external validation on DACHS for MSI and *BRAF* status prediction are listed in Tables S3–S8.

### SSL-attMIL is domain-shift invariant

Domain shifts can still hide behind high AUROC values and can severely limit the real-world performance of DL models.^38^ We investigated the distribution of the image-only Wang-attMIL model prediction scores for MSI and *BRAF* in the training and test cohort. We found that the prediction scores were similarly distributed in the training and test set for the image-only Wang-attMIL (Figure S4) as well as for the multi-input models (Figure S5). In summary, these data show that Wang-attMIL yields classifiers with high generalizability across the two datasets, which are independent of Macenko normalization and do not display domain shifts. Furthermore, adding clinical data to the models leads to even better generalization.

### Attention-based models attend to relevant tissue regions

**Figure 4:**
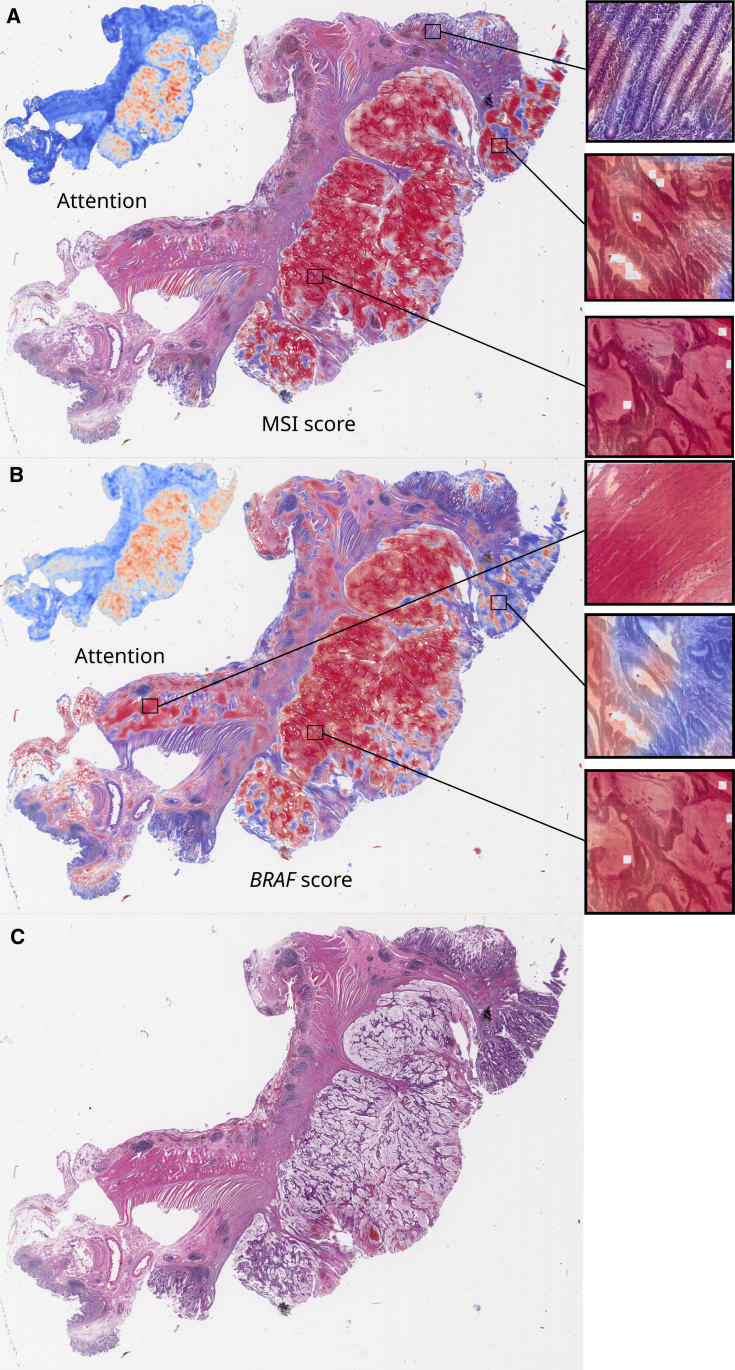
Spatial patterns of attention and classification of MSI and *BRAF* prediction models (A and B) MSI score (A) and *BRAF* score (B) with corresponding attention maps for a typical MSI- and *BRAF*-positive patient from the DACHS cohort. (C) Plain slide view. Scores were obtained with the best in-domain models trained on QUASAR (Wang-attMIL model). The displayed attention distribution is the normalized attention $â=\frac{a-a_{\min}}{a_{\max}-a_{\min}}$, where a is the attention score and $a_{\min}$ and $a_{\max}$ are the minimum and maximum scores on the WSI. This attention map highlights “relevant” tumor regions, irrespective of whether they were predicted to be MSI or MSS. The classification scores of the model show the “MSI-ness” and “*BRAF*-ness” for each tile. In both cases, the model correctly predicted MSI and BRAF status on the patient level.

To comprehend the decision-making processes of trained DL models, we investigated the visual patterns in their spatial context on WSIs. We separately visualized attention and prediction heatmaps for typical patients for the image-only Wang-attMIL models (Figures 4A and 4B). For MSI prediction, high-attention regions were confined to the tumor tissue, while fibromuscular tissue and non-tumor epithelium were not attended to as much by the model (Figures 4A and 4B). In *BRAF* prediction, however, the attention was more spread out. Tumor tissue is still attended more to than non-tumor tissue but to a lesser extent (Figures 4A and 4B). This indicates that either the *BRAF* prediction model did not learn to focus sufficiently on the tumor tissue or that the *BRAF* prediction model learned that visual features outside of the tumor region are somewhat relevant to making predictions. In particular, lymphocyte-infiltrated muscle tissue was assigned a high *BRAF* and attention score. Confounding factors in images for *BRAF* status prediction are yet another possibility. Further high-resolution heatmaps for MSI and *BRAF* status for typical patients are available at Zenodo: https://doi.org/10.5281/zenodo.7454743. Interestingly, the presence of pen marks on some slides did not confuse the models, as pen marks were assigned a very low attention score, showing that the image-only Wang-attMIL model is very robust, even to the presence of artifacts.

### Distinct visual features drive MSI and *BRAF* prediction

**Figure 5:**
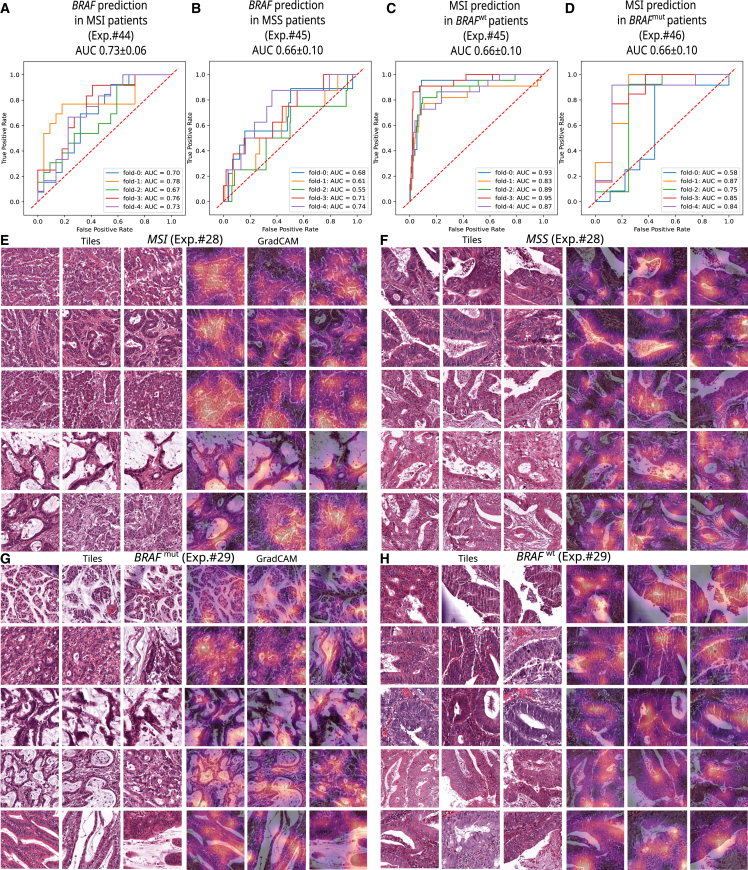
Biomarker predictability in patient subgroups and explainability (A) Internal validation ROCs for *BRAF* mutation prediction in the subgroup of MSI patients. (B) Internal validation ROCs for *BRAF* status prediction in the subgroup of MSS patients. (C) Internal validation ROCs for MSI/MSS status prediction in the subgroup of *BRAF*-mutated patients. (D) Internal validation ROCs for MSI/MSS status prediction in the subgroup of *BRAF* wild-type patients. (E and F) Top scoring tiles and Grad-CAM saliency maps for MSI (E) and MSS (F) status for the best in-domain Wang+attMIL model deployed on the DACHS cohort. (G and H) Top scoring tiles for *BRAF*-mutated (G) and *BRAF* wild-type (H) status for the best in-domain Wang+attMIL model deployed on the DACHS cohort. For better interpretability, six out-of-focus tiles are not shown in this panel. In (E)–(G), top tiles are the highest, top 5%, and top 10% scoring tiles in terms of the product of the tile’s attention and the tile’s classification score (left to right) for the patients with the highest overall classification score for the target mutation (top to bottom). High-resolution images can be found at Zenodo: https://doi.org/10.5281/zenodo.7454743. Correlation of prediction scores for MSI and *BRAF* status for the best image-only model can be found in Figure S6.

MSI and *BRAF* mutant status are highly correlated; therefore, we addressed whether the models recognize different sets of visual features for either target. First, we investigated whether *BRAF* mutations can be predicted in the MSI and microsatellite stable (MSS) subgroups of the QUASAR trial dataset. Using image-only Wang-attMIL models, the DL system was able to detect *BRAF* mutational status in the MSI subgroup, reaching a cross-validated AUROC of 0.73 ± 0.06 (Figure 5A). However, *BRAF* status was not predictable in the MSS subgroup, reaching an AUROC of 0.66 ± 0.10 (Figure 5B). Second, we repeated the analysis for MSI status prediction in *BRAF*-mutated and wild-type subgroups in analogy: MSI status was predictable in *BRAF*^wt^ patients (AUROC 0.89 ± 0.06, Figure 5C) and *BRAF*^mut^ patients (AUROC 0.78 ± 0.15, Figure 5D). We further investigated the visual features present in image tiles that were assigned high attention and a high-class prediction score at the same time. We found that MSI (Figure 5E) and MSS (Figure 5F) tiles showed similar patterns to those described previously: poorly differentiated tumor glands with immune-infiltrated stroma in MSI versus well-differentiated stroma-rich tissue areas for MSS.^17^^,^^24^ *BRAF*^mut^ (Figure 5G) and *BRAF*^wt^ (Figure 5H) top tiles showed different prominent patterns than MSI and MSS tiles, with mucinous differentiation dominating *BRAF*^mut^ tiles and well-differentiated, stroma-rich patterns dominating *BRAF*^wt^ tiles. Using gradient-weighted class activation mapping (Grad-CAM) to highlight relevant subregions in these top tiles, we found that the models indeed focused on these tissue structures (Figures 5E–5H). MSI and *BRAF* prediction scores were correlated in all patient subgroups (Figure S6). Taken together, these data show that MSI and *BRAF* prediction models detect distinct visual features that are compatible with previous knowledge; however, MSI features appear to be more distinct, as MSI status is easier detectable in subgroups of *BRAF*^mut^/*BRAF*^wt^ than *BRAF* status in subgroups of MSI/MSS.

## Discussion

MSI prediction from histopathology with DL has been investigated since 2019.^7^^,^^14^^,^^17^^,^^22^ Earlier works used the INPT approach using mean pooling for slide-level aggregation.^7^ Recent studies have investigated attention-based MIL approaches in the hope of less noisy supervision and creating models able to learn to combine global features.^39^^,^^40^^,^^41^^,^^42^ Most recently, SSL methods have been adopted in the histopathology domain.^12^ In a smaller pilot study, the attMIL approach has shown superior performance compared with the INPT approach.^12^ The main limitations of many of these works, however, are that (1) they focus on only a few clinically relevant tasks and (2) they are not validated on external cohorts, thus lacking performance evaluation in realistic scenarios. First, we tested the performance of two attMIL-models with different pre-trained encoders on multiple clinically relevant biomarkers. Second, we investigated their external validation performance on a large dataset for internally well-predictable biomarkers. For the attMIL approach, this degree of large-scale validation is required for clinical translation but was missing from previous studies.^23^

This study evaluates current state-of-the-art methods for biomarker prediction in CRC from pathology slides in a realistic evaluation setting: SSL-attMIL with the Wang encoder outperformed all other approaches. This confirms the superiority of the attMIL approach when combined with an appropriate encoder on a large external dataset. Our Wang-attMIL models were generalizable and invariant to the color normalization in the test set. In contrast, this was not the case for our Ciga-attMIL models, where the encoder was trained on a similar, but much smaller, dataset compared with the Wang encoder. This provides empirical evidence that Wang’s encoder trained via the clustering-guided contrastive learning (CCL) algorithm is superior to Ciga’s encoder trained via SimCLR for the biomarker prediction investigated in this article. Thus, the Wang encoder provides an ideal backbone for the attMIL approach for biomarker prediction at hand. Using the image-only Wang-attMIL models, our approach improves the AUROC for MSI prediction from 0.68 to 0.92 for training on QUASAR and testing on DACHS compared with Echle et al.^9^ These results are in line with previous studies, which demonstrated the superiority of the attMIL approach for biomarker prediction.^12^^,^^22^^,^^40^^,^^43^

Further, we demonstrated that morphological features most relevant for a prediction made by our best image-only MSI and *BRAF* models are in line with previous findings and pathological knowledge.^17^^,^^24^^,^^25^ In addition, the current study extends these previous findings by (1) showing the superiority of the Wang-attMIL models using large cohorts with thousands of patients and (2) investigating multiple biomarkers beyond MSI.

Finally, we tested extensions of the image-only Wang-attMIL model by concatenating image vectors with vectors representing clinical patient data. Here, we did not see direct synergy in performance on the QUASAR cohort, but we did see enhanced prediction performance for patients in the DACHS cohort. This is true in particular for the prediction of *BRAF* biomarker status, which shows a weaker morphological phenotype compared with MSI mutations. In this case, multi-input models stabilized predictions across different datasets.

Prediction of genetic alterations such as MSI and *BRAF* mutation is regarded as one of the most relevant applications of computational pathology.^2^ Exceeding pure research applications, the prediction of MSI status has enormous commercial potential. This is evident in multiple companies that have developed solutions for MSI status prediction,^43^^,^^44^^,^^45^ one of which has received CE/IVD regulatory approval in Europe in 2022 (“MSIntuit CRC” by Owkin). Here, we chose to make our technology publicly available under an MIT open-source license so that anyone can re-use it.

### Limitations of the study

However, we also identified limitations of DL-based biomarker prediction. While previous studies have suggested that mutations in *KRAS*, *NRAS*, and *PIK3CA* might be predictable from pathology images,^4^^,^^22^^,^^46^ we show that this performance is not in a clinically relevant range with the methods described in this article. Although prediction of these biomarkers was possible with non-random AUROCs above 0.5, this is far from suitable for clinical application. Also, we show that a trivial model that uses only age, gender, organ, and sidedness as an input reaches similar performances for the prediction of *NRAS* and *PIK3CA* genes (Table 1). Thereby, our study provides suggestive evidence that despite the use of large, multi-centric patient cohorts and powerful DL models, it is not possible to predict the mutational status of *KRAS*, *NRAS*, and *PIK3CA* from CRC histopathology slides with current methods.

## STAR★Methods

### Key resources table

**Table:** 

REAGENT or RESOURCE	SOURCE	IDENTIFIER
**Software and algorithms**		

deepmed	GitHub: https://github.com/KatherLab/deepmed	N/A
marugoto	GitHub: https://github.com/KatherLab/marugoto	N/A
Wang’s encoder	GitHub: https://github.com/Xiyue-Wang/RetCCL	https://doi.org/10.1016/j.media.2022.102645
Ciga’s encoder	GitHub: https://github.com/ozanciga/self-supervised-histopathology	https://doi.org/10.1016/j.mlwa.2021.100198

**Deposited data**		

heatmaps and high-resolution top tiles	Zenodo: https://doi.org/10.5281/zenodo.7454743	https://doi.org/10.5281/zenodo.7454743
trained models	GitHub: https://github.com/KatherLab/crc-models-2022	this manuscript

**Other**		

GPU Quadro RTX 8000	Nvidia Corp., Santa Clara, California.	N/A

### Resource availability

#### Lead contact

Requests for further information on software and resources should be directed and will be fulfilled by the lead contact, Jakob N. Kather (jakob-nikolas.kather@alumni.dkfz.de).

#### Materials availability

This study did not generate new unique reagents.

### Experimental model and subject details

#### Ethics statement

This study was performed in accordance with the Declaration of Helsinki. This study is a retrospective analysis of digital images of anonymized archival tissue samples of multiple cohorts of CRC patients. Data were collected and anonymized and ethical approval was obtained. The use of tissue samples from QUASAR^47^ was approved by the North East – York Research Ethics Committee (08/H0903/62). DACHS was approved by the Ethics committee of the Medical Faculty at Heidelberg University (310/2001).^48^

#### Patient cohorts

QUASAR is the “Quick and Simple and Reliable” trial (Yorkshire, UK), which investigated treatment efficacy in patients from the United Kingdom with mostly stage II colorectal tumors.^47^^,^^49^ DACHS (Darmkrebs: Chancen der Verhütung durch Screening, Southwest Germany)^50^^,^^51^ is a population-based case-control and patient cohort study on CRC including samples from patients of all tumor stages (I-IV) collected from different laboratories in the south-west of Germany coordinated by the German Cancer Research Center (Heidelberg, Germany). In QUASAR, mismatch-repair deficiency (dMMR) or proficiency (pMMR) was determined with immunohistochemistry on tissue microarrays (two-plex for MLH1 and MSH2).^21^ Mutational data for *BRAF*, *KRAS*, *NRAS,* and PIK3CA was obtained via pyrosequencing.^52^ In DACHS, MSI status was determined with a three-plex PCR assay using the mononucleotide markers BAT25, BAT26, and CAT25 in tissue sections of the paraffin-embedded tumor block. In previous work, this marker panel was shown to differentiate MSI-high from non-MSI-high tumors with a 100% concordance of MSI-high tumors compared with the National Cancer Institute/International Collaborative Group on HNPCC (NCI/ICG-HNPCC) marker panel, which includes the five markers BAT25, BAT26, D17S250, D2S123, and D5S346.^53^^,^^54^ Mutational data for KRAS and BRAF was obtained by various methods in subsets of this multicenter study. In detail, the methods were the single-stranded conformational polymorphism technique and immunohistochemical analyses,^55^ respectively, or by Sanger sequencing.^56^ CONSORT charts with details on missing data and preprocessing drop out for the QUASAR and DACHS cohort can be found in Figures S1 and S2.

### Method details

#### Image preprocessing

All images from H&E stained resection tissue slides were preprocessed according to the “Aachen protocol for deep learning histopathology”.^57^ WSIs were tessellated into 512x512 pixels image tiles of 256 μm edge length. Tissue regions were automatically selected using RGB thresholding (summed median brightness across RGB channels < 660) and canny edge detection by requiring at least four edges per image tile.^40^ All remaining tiles were included in the analysis. The fraction of blurry or homogenous tiles was estimated using the method of variation of the Laplacian,^58^ which showed that 9.2% and 3.4% of the tiles stayed below a score value of 80 in the QUASAR and DACHS cohorts, respectively. Tiles were processed at 224 px edge length (effective resolution of 1.14 μm per pixel) using bilinear interpolation as implemented in PyTorch’s “Resize” function and normalized with ImageNet’s mean and standard deviation of RGB pixel values. Tiles in the training set were color-normalized with Macenko’s method using a reference image tile.^7^^,^^59^ In the test set, the performance of models was assessed in color-normalized and native tiles.

#### Biomarker prediction from whole slide images

We compare results obtained with two different DL approaches – the INPT approach against the attMIL approach. Both approaches address a classification problem in which the objective is to predict a slide label from a collection of individual tiles.

In the INPT approach,^7^^,^^30^ a DL network pre-trained on ImageNet is fine-tuned using the WSI-level label assigned to each tumor tile. Slide-level predictions are then obtained by averaging/mean-pooling of tile-level predictions. This has resulted in high-performance models,^9^ but imperfect generalization to external cohorts.^21^

The attMIL approach is a two-stage process: First, images of tiles are compressed to image feature vectors using a pre-trained encoder network. Second, the image feature vectors are used as input to a network that uses an attention mechanism for aggregation of predictions from tile to slide level. In short, this network computes an attention-weighted average of the input feature vectors which is then classified and can thus learn which parts of the input image should be discarded for the final prediction. We trained and tested models on top of two publicly available frozen encoders trained with self-supervised learning (SSL), referring to the generic pipeline as “SSL-attMIL”. Ciga et al. applied SimCLR^60^ to train a ResNet-18 on 400,000 pathology images selected from 57 datasets.^61^ Wang et al. trained a ResNet-50 on a total of 15 million pathology images retrieved from 32,000 WSIs from the full TCGA and PAIP dataset via a clustering-guided contrastive learning (CCL) SSL algorithm.^62^ In CCL, the learning objective is to minimize the contrastive loss between any two tiles from the same WSI and to maximize the loss for any two tiles from different WSIs.^62^ In SimCLR, the contrastive loss is minimized for the same tile and maximized between any two different tiles.^60^ We used both pre-trained models to extract 1024 (“Ciga-attMIL”) and 2048 (“Wang-attMIL”) features per tile. The set of features from all or a large subset of tiles from a WSI (we randomly sampled 512 every epoch per WSI) was then used as input to the basic attMIL model^32^ that learns to predict a single label for a WSI.

Finally, we extended the basic attMIL approach by adding basic clinicopathological data as an additional input to the model. These input data are known to be associated with MSI status:^24^ gender, age, tumor sidedness (lef/right) and organ (colon/rectum) (Table 2). To this end, each patient’s clinical data was embedded into a vector representation. For each tile, this clinical data vector was concatenated with the image feature vector.

Setting all values of the image feature vectors to zero results in yet another model that solely depends on clinical data. We call the two described model architectures the “multi-input” and “solely clinical-data-based” models. The multi-input and solely clinical-data-based models were trained using the same hyperparameters as in the image-only approach. Detailed information on the training procedure and model details are available in the STAR Methods.

#### Visualization and explainability

Visualization of important morphological features relevant to the decision-making processes of DL models is important for: 1) Finding if there are distinct morphologies for various mutations and 2) better comprehension of model internals. For visualization, we used three approaches. We showed the highest-scoring tiles from patients that are correctly classified with the highest scores.^63^ Additionally, we apply Grad-CAM,^64^ a generalization of the class activation mapping (CAM) algorithm.^65^ Finally, WSI heatmaps display separate spatial distributions of the attention and prediction scores.

#### Implementation of the INPT approach

In our implementation, tiles were direct inputs for transfer learning. Transfer learning requires a convolutional neural network (ResNet-18) that was pre-trained on ImageNet combined with appropriate substitution for the fully connected classification head. First, the new head’s weights are trained with all other layers’ weights frozen; subsequently, the remaining layers' weights are unfrozen and fine-tuned. Thus the network learns to predict the biomarker status for a single tile, and the patient score is calculated by averaging across all tiles for a given patient. We used our in-house open-source pipeline DeepMed^66^ with a batch size of 92, the Adam optimizer ($\beta_{1}=0.9,\beta_{2}=0.99,\varepsilon =10^{-5}$), and a learning rate of 2e-3 and 1% weight decay.^67^ The cross-entropy loss function was weighted by the inverse of class frequencies to account for class imbalances. After fine-tuning the model’s head for one epoch, the full model was trained for 32 epochs during which the learning rate was scheduled by a modified “1 cycle policy” as made available by fastAI^68^^,^^69^^,^^70^ Maximum learning rates were set in equally spaced slices from lr_max=1e-3 for the deepest layer to lr_max/100 for the shallowest layer, respectively. The learning rates sinusoidally increased from 1/5 of the maxima to the maxima over ten epochs. Then, the learning rates were sinusoidally decreased from the maxima to 1/10,000 of the maxima over the remaining epochs. At the same time, $\beta_{1}$ was sinusoidally varied from 0.95 to 0.85 over the first ten and back to 0.95 over the remaining epochs. During training, tiles in the training data set were augmented by combined operations of random rotations up to 360° with 75 % and vertical flips with 50 % probability.

#### Implementation of attention-based multiple instance learning

In both self-supervised learning-attMIL approaches, a fully connected layer followed by ReLU embeds the features in a 256-dimensional space. This embedded vector is then passed through a linear layer that outputs another 256-dimensional vector $h_{k}$ for tile k. Then the attention score $a_{k}$ for the k-th tile is calculated via:

(Equation 1) $$a_{k}=\frac{\exp\left\{w^{T}\;\tanh\left({Vh}_{k}\right)\right\}}{\sum_{j=1}^{K}\exp\left\{w^{T}\;\tanh\left({Vh}_{j}\right)\right\}}$$

where $h\in R^{256},V\in R^{128x256}$, $w\in R^{128}$ and K is the maximal number of tiles randomly resampled every epoch for each patient. Then the MIL pooling operation is applied via:

(Equation 2) $$h_{sum}=\sum_{i=1}^{K}a_{i}h_{i}\text{,}$$

where $h_{i}$ is the i-th tile’s embedding; a maximum of K =512 tiles were used per patient. To obtain the final probability score for each patient, the batch of $h_{sum}$ s is passed through a BatchNorm1D layer, followed by Dropout layer with p=50%. Then, $h_{sum}$ is passed through a fully connected layer with two output dimensions and finally, a softmax layer is applied to obtain the scores. The batch size was 32 patients, the number of epochs was 32, the maximal learning rate was sinusoidally varied from lr_max/25 to lr_max=1e-4 over eight epochs and back to lr_max/10.000 over the remaining epochs, no learning rate slicing was applied, $\beta_{1}$ was varied with the same periodicity, and other hyperparameters were the same as in the INPT approach.

#### Implementation of multi-input prediction models

We one-hot encoded the patient’s gender and tumor location and added the age (years) as an integer variable. All variables were normalized to be zero-centered with a normal distribution. Missing values were filled using mean-imputation. These features were concatenated with a tile’s image feature vector before training. This extended vector was then used as input to the attMIL approach. We performed an ablation study by setting the image features to zero to test the performance of a solely clinical-data-based model separately.

### Quantification and statistical analysis

#### Experimental design and statistics

We trained all neural network models on QUASAR via stratified five-fold cross-validation on the level of patients (“within-cohort experiment”, for MSI, *BRAF*, *KRAS*, *NRAS,* and *PIK3CA*). Subsequently, we applied all five models to the external validation cohort DACHS (only for MSI and *BRAF*). During cross-validation, a validation subset (25% of the training data) was randomly split off every training set to check for overfitting. The area under the receiver operator characteristics curve (AUROC) and the area under the precision-recall curve (AUPRC) give statistical endpoints in our analysis, the latter being more robust to class imbalance. For clarity, we numbered all of our experiments and summarized the results in Table 1. AUROCs of trained models for internal and for external validation for MSI and *BRAF* status prediction on DACHS are compared using the analysis of variances (ANOVA) test and p-values are listed in Tables S3–S8. In addition to the AUROC, we evaluated the sensitivity and specificity of our models at thresholds of 0.25, 0.5, 0.75, and a threshold giving a 95% in-domain sensitivity. The 95% in-domain sensitivity threshold was obtained by taking the average of each model’s 95% sensitivity thresholds on its respective internal test dataset.

## Data Availability

The DACHS and QUASAR data used in this study cannot be deposited in a public repository because of local ethical prohibitions. All source codes are available at GitHub: https://github.com/KatherLab/marugoto. Heatmaps for typical patients and high-resolution images of top tiles have been deposited at Zenodo at Zenodo: https://doi.org/10.5281/zenodo.7454743. Models trained in this study have been deposited to GitHub: https://github.com/KatherLab/crc-models-2022. Any additional information required to reanalyze the data reported in this paper is available from the lead contact upon request.

## Supplemental information

Document S1. Tables S1–S8 and Figures S1–S6

Document S2. Article plus supplemental information

## References

[1] Esteva A., Kuprel B., Novoa R.A., Ko J., Swetter S.M., Blau H.M., Thrun S. (2017). Dermatologist-level classification of skin cancer with deep neural networks. Nature.

[2] Heinz C.N., Echle A., Foersch S., Bychkov A., Kather J.N. (2022). The future of artificial intelligence in digital pathology - results of a survey across stakeholder groups. Histopathology.

[3] Echle A., Rindtorff N.T., Brinker T.J., Luedde T., Pearson A.T., Kather J.N. (2021). Deep learning in cancer pathology: a new generation of clinical biomarkers. Br. J. Cancer.

[4] Cifci D., Foersch S., Kather J.N. (2022). Artificial intelligence to identify genetic alterations in conventional histopathology. J. Pathol..

[5] Chen R.J., Lu M.Y., Williamson D.F.K., Chen T.Y., Lipkova J., Noor Z., Shaban M., Shady M., Williams M., Joo B., Mahmood F. (2022). Pan-cancer integrative histology-genomic analysis via multimodal deep learning. Cancer Cell.

[6] Bera K., Schalper K.A., Rimm D.L., Velcheti V., Madabhushi A. (2019). Artificial intelligence in digital pathology - new tools for diagnosis and precision oncology. Nat. Rev. Clin. Oncol..

[7] Kather J.N., Pearson A.T., Halama N., Jäger D., Krause J., Loosen S.H., Marx A., Boor P., Tacke F., Neumann U.P. (2019). Deep learning can predict microsatellite instability directly from histology in gastrointestinal cancer. Nat. Med..

[8] Cao R., Yang F., Ma S.-C., Liu L., Zhao Y., Li Y., Wu D.-H., Wang T., Lu W.-J., Cai W.-J. (2020). Development and interpretation of a pathomics-based model for the prediction of microsatellite instability in Colorectal Cancer. Theranostics.

[9] Echle A., Grabsch H.I., Quirke P., van den Brandt P.A., West N.P., Hutchins G.G.A., Heij L.R., Tan X., Richman S.D., Krause J. (2020). Clinical-grade detection of microsatellite instability in colorectal tumors by deep learning. Gastroenterology.

[10] Bilal M., Ahmed Raza S.E., Azam A., Graham S., Ilyas M., Cree I.A., Snead D., Minhas F., Rajpoot N.M. (2021).

[11] Lee S.H., Song I.H., Jang H.-J. (2021). Feasibility of deep learning-based fully automated classification of microsatellite instability in tissue slides of colorectal cancer. Int. J. Cancer.

[12] Schirris Y., Gavves E., Nederlof I., Horlings H.M., Teuwen J. (2022). DeepSMILE: contrastive self-supervised pre-training benefits MSI and HRD classification directly from H&E whole-slide images in colorectal and breast cancer. Med. Image Anal..

[13] Schrammen P.L., Ghaffari Laleh N., Echle A., Truhn D., Schulz V., Brinker T.J., Brenner H., Chang-Claude J., Alwers E., Brobeil A. (2022). Weakly supervised annotation-free cancer detection and prediction of genotype in routine histopathology. J. Pathol..

[14] Yamashita R., Long J., Longacre T., Peng L., Berry G., Martin B., Higgins J., Rubin D.L., Shen J. (2021). Deep learning model for the prediction of microsatellite instability in colorectal cancer: a diagnostic study. Lancet Oncol..

[15] Jang H.-J., Lee A., Kang J., Song I.H., Lee S.H. (2020). Prediction of clinically actionable genetic alterations from colorectal cancer histopathology images using deep learning. World J. Gastroenterol..

[16] Kather J.N., Calderaro J. (2020). Development of AI-based pathology biomarkers in gastrointestinal and liver cancer. Nat. Rev. Gastroenterol. Hepatol..

[17] Echle A., Laleh N.G., Schrammen P.L., West N.P., Trautwein C., Brinker T.J., Gruber S.B., Buelow R.D., Boor P., Grabsch H.I. (2021). Deep learning for the detection of microsatellite instability from histology images in colorectal cancer: a systematic literature review. ImmunoInformatics.

[18] Mlecnik B., Bindea G., Angell H.K., Maby P., Angelova M., Tougeron D., Church S.E., Lafontaine L., Fischer M., Fredriksen T. (2016). Integrative analyses of colorectal cancer show immunoscore is a stronger predictor of patient survival than microsatellite instability. Immunity.

[19] Yoon H.H., Jin Z., Kour O., Kankeu Fonkoua L.A., Shitara K., Gibson M.K., Prokop L.J., Moehler M., Kang Y.-K., Shi Q., Ajani J.A. (2022). Association of PD-L1 expression and other variables with benefit from immune checkpoint inhibition in advanced gastroesophageal cancer: systematic review and meta-analysis of 17 phase 3 randomized clinical trials. JAMA Oncol..

[20] Poston G.J., Tait D., O’Connell S., Bennett A., Berendse S., Guideline Development Group (2011). Diagnosis and management of colorectal cancer: summary of NICE guidance. BMJ.

[21] Echle A., Ghaffari Laleh N., Quirke P., Grabsch H.I., Muti H.S., Saldanha O.L., Brockmoeller S.F., van den Brandt P.A., Hutchins G.G.A., Richman S.D. (2022). Artificial intelligence for detection of microsatellite instability in colorectal cancer—a multicentric analysis of a pre-screening tool for clinical application. ESMO Open.

[22] Bilal M., Raza S.E.A., Azam A., Graham S., Ilyas M., Cree I.A., Snead D., Minhas F., Rajpoot N.M. (2021). Development and validation of a weakly supervised deep learning framework to predict the status of molecular pathways and key mutations in colorectal cancer from routine histology images: a retrospective study. Lancet. Digit. Health.

[23] Kleppe A., Skrede O.-J., De Raedt S., Liestøl K., Kerr D.J., Danielsen H.E. (2021). Designing deep learning studies in cancer diagnostics. Nat. Rev. Cancer.

[24] Greenson J.K., Huang S.-C., Herron C., Moreno V., Bonner J.D., Tomsho L.P., Ben-Izhak O., Cohen H.I., Trougouboff P., Bejhar J. (2009). Pathologic predictors of microsatellite instability in colorectal cancer. Am. J. Surg. Pathol..

[25] Pai R.K., Jayachandran P., Koong A.C., Chang D.T., Kwok S., Ma L., Arber D.A., Balise R.R., Tubbs R.R., Shadrach B., Pai R.K. (2012). BRAF-Mutated, microsatellite-stable adenocarcinoma of the proximal colon. Am. J. Surg. Pathol..

[26] Rosner A., Miyoshi K., Landesman-Bollag E., Xu X., Seldin D.C., Moser A.R., MacLeod C.L., Shyamala G., Gillgrass A.E., Cardiff R.D. (2002). Pathway pathology: histological differences between ErbB/Ras and Wnt pathway transgenic mammary tumors. Am. J. Pathol..

[27] Hewitt L.C., Saito Y., Wang T., Matsuda Y., Oosting J., Silva A.N.S., Slaney H.L., Melotte V., Hutchins G., Tan P. (2019). KRAS status is related to histological phenotype in gastric cancer: results from a large multicentre study. Gastric Cancer.

[28] Howard F.M., Dolezal J., Kochanny S., Schulte J., Chen H., Heij L., Huo D., Nanda R., Olopade O.I., Kather J.N. (2021). The impact of site-specific digital histology signatures on deep learning model accuracy and bias. Nat. Commun..

[29] Shmatko A., Ghaffari Laleh N., Gerstung M., Kather J.N. (2022). Artificial intelligence in histopathology: enhancing cancer research and clinical oncology. Nat. Cancer.

[30] Coudray N., Ocampo P.S., Sakellaropoulos T., Narula N., Snuderl M., Fenyö D., Moreira A.L., Razavian N., Tsirigos A. (2018). Classification and mutation prediction from non–small cell lung cancer histopathology images using deep learning. Nat. Med..

[31] Fu Y., Jung A.W., Torne R.V., Gonzalez S., Vöhringer H., Shmatko A., Yates L.R., Jimenez-Linan M., Moore L., Gerstung M. (2020). Pan-cancer computational histopathology reveals mutations, tumor composition and prognosis. Nat. Cancer.

[32] Ilse M., Tomczak J.M., Welling M. (2018). Attention-based deep multiple instance learning. arXiv.

[33] Saldanha O.L., Loeffler C.M.L., Niehues J.M., van Treeck M., Seraphin T.P., Hewitt K.J., Cifci D., Veldhuizen G.P., Ramesh S., Pearson A.T. (2022). Self-supervised deep learning for pan-cancer mutation prediction from histopathology. bioRxiv.

[34] Seraphin T.P., Luedde M., Roderburg C., van Treeck M., Schneider P., Buelow R.D., Boor P., Loosen S., Provaznik Z., Mendelsohn D. (2022). Prediction of heart transplant rejection from routine pathology slides with self-supervised Deep Learning. medRxiv.

[35] Howard F.M., Kather J.N., Pearson A.T. (2023). Multimodal deep learning: an improvement in prognostication or a reflection of batch effect?. Cancer Cell.

[36] Mandrekar J.N. (2010). Receiver operating characteristic curve in diagnostic test assessment. J. Thorac. Oncol..

[37] Campanella G., Hanna M.G., Geneslaw L., Miraflor A., Werneck Krauss Silva V., Busam K.J., Brogi E., Reuter V.E., Klimstra D.S., Fuchs T.J. (2019). Clinical-grade computational pathology using weakly supervised deep learning on whole slide images. Nat. Med..

[38] Kleppe A. (2022). Area under the curve may hide poor generalisation to external datasets. ESMO Open.

[39] Zeng Q., Klein C., Caruso S., Maille P., Laleh N.G., Sommacale D., Laurent A., Amaddeo G., Gentien D., Rapinat A. (2022). Artificial intelligence predicts immune and inflammatory gene signatures directly from hepatocellular carcinoma histology. J. Hepatol..

[40] Ghaffari Laleh N., Muti H.S., Loeffler C.M.L., Echle A., Saldanha O.L., Mahmood F., Lu M.Y., Trautwein C., Langer R., Dislich B. (2022). Benchmarking weakly-supervised deep learning pipelines for whole slide classification in computational pathology. Med. Image Anal..

[41] Lipkova J., Chen T.Y., Lu M.Y., Chen R.J., Shady M., Williams M., Wang J., Noor Z., Mitchell R.N., Turan M. (2022). Deep learning-enabled assessment of cardiac allograft rejection from endomyocardial biopsies. Nat. Med..

[42] Lu M.Y., Chen T.Y., Williamson D.F.K., Zhao M., Shady M., Lipkova J., Mahmood F. (2021). AI-based pathology predicts origins for cancers of unknown primary. Nature.

[43] Saillard C., Dehaene O., Marchand T., Moindrot O., Kamoun A., Schmauch B., Jegou S. (2021). Self supervised learning improves dMMR/MSI detection from histology slides across multiple cancers. arXiv.

[44] Joshi R.P., Kruger A.J., Sha L., Kannan M., Khan A.A., Stumpe M. (2020). Learning relevant H&E slide morphologies for prediction of colorectal cancer tumor mutation burden using weakly supervised deep learning. J. Clin. Orthod..

[45] Arslan S., Mehrotra D., Schmidt J., Geraldes A., Singhal S., Hense J., Li X., Bass C., Kather J.N., Raharja-Liu P. (2022). Deep learning can predict multi-omic biomarkers from routine pathology images: a systematic large-scale study. bioRxiv.

[46] Kather J.N., Heij L.R., Grabsch H.I., Loeffler C., Echle A., Muti H.S., Krause J., Niehues J.M., Sommer K.A.J., Bankhead P. (2020). Pan-cancer image-based detection of clinically actionable genetic alterations. Nat. Cancer.

[47] Gray R., Barnwell J., McConkey C., Hills R.K., Williams N.S., Kerr D.J., Quasar Collaborative Group (2007). Adjuvant chemotherapy versus observation in patients with colorectal cancer: a randomised study. Lancet.

[48] Brenner H., Chang-Claude J., Seiler C.M., Stürmer T., Hoffmeister M. (2006). Does a negative screening colonoscopy ever need to be repeated?. Gut.

[49] Quirke P., Morris E. (2007). Reporting colorectal cancer. Histopathology.

[50] Brenner H., Chang-Claude J., Seiler C.M., Hoffmeister M. (2011). Long-term risk of colorectal cancer after negative colonoscopy. J. Clin. Oncol..

[51] Hoffmeister M., Jansen L., Rudolph A., Toth C., Kloor M., Roth W., Bläker H., Chang-Claude J., Brenner H. (2015). Statin use and survival after colorectal cancer: the importance of comprehensive confounder adjustment. J. Natl. Cancer Inst..

[52] Hutchins G., Southward K., Handley K., Magill L., Beaumont C., Stahlschmidt J., Richman S., Chambers P., Seymour M., Kerr D. (2011). Value of mismatch repair, KRAS, and BRAF mutations in predicting recurrence and benefits from chemotherapy in colorectal cancer. J. Clin. Oncol..

[53] Boland C.R., Thibodeau S.N., Hamilton S.R., Sidransky D., Eshleman J.R., Burt R.W., Meltzer S.J., Rodriguez-Bigas M.A., Fodde R., Ranzani G.N., Srivastava S. (1998). A National Cancer Institute Workshop on Microsatellite Instability for cancer detection and familial predisposition: development of international criteria for the determination of microsatellite instability in colorectal cancer. Cancer Res..

[54] Findeisen P., Kloor M., Merx S., Sutter C., Woerner S.M., Dostmann N., Benner A., Dondog B., Pawlita M., Dippold W. (2005). T25 repeat in the 3’ untranslated region of the CASP2 gene: a sensitive and specific marker for microsatellite instability in colorectal cancer. Cancer Res..

[55] Bläker H., Helmchen B., Bönisch A., Aulmann S., Penzel R., Otto H.F., Rieker R.J. (2004). Mutational activation of the RAS-RAF-MAPK and the Wnt pathway in small intestinal adenocarcinomas. Scand. J. Gastroenterol..

[56] Jia M., Jansen L., Walter V., Tagscherer K., Roth W., Herpel E., Kloor M., Bläker H., Chang-Claude J., Brenner H., Hoffmeister M. (2016). No association of CpG island methylator phenotype and colorectal cancer survival: population-based study. Br. J. Cancer.

[57] Muti H.S., Loeffler C., Echle A., Heij L.R., Buelow R.D., Krause J., Broderius L., Niehues J., Liapi G., Boor P. (2020).

[58] Pech-Pacheco J.L., Cristobal G., Chamorro-Martinez J., Fernandez-Valdivia J. (2000). Proceedings 15th International Conference on Pattern Recognition. ICPR-2000.

[59] Macenko M., Niethammer M., Marron J.S., Borland D., Woosley J.T., Guan X., Schmitt C., Thomas N.E. (2009). 2009 IEEE International Symposium on Biomedical Imaging: From Nano to Macro.

[60] Chen T., Kornblith S., Norouzi M., Hinton G. (2020). A Simple framework for contrastive learning of visual representations. arXiv.

[61] Ciga O., Xu T., Martel A.L. (2022). Self supervised contrastive learning for digital histopathology. Mach. Learn. Appl..

[62] Wang X., Du Y., Yang S., Zhang J., Wang M., Zhang J., Yang W., Huang J., Han X. (2023). RetCCL: clustering-guided contrastive learning for whole-slide image retrieval. Med. Image Anal..

[63] Brockmoeller S., Echle A., Ghaffari Laleh N., Eiholm S., Malmstrøm M.L., Plato Kuhlmann T., Levic K., Grabsch H.I., West N.P., Saldanha O.L. (2022). Deep Learning identifies inflamed fat as a risk factor for lymph node metastasis in early colorectal cancer. J. Pathol..

[64] Selvaraju R.R., Cogswell M., Das A., Vedantam R., Parikh D., Batra D. (2017). Grad-cam: visual explanations from deep networks via gradient-based localization. Proc. Est. Acad. Sci. Biol. Ecol..

[65] Zhou B., Khosla A., Lapedriza A., Oliva A., Torralba A. (2016). Lapedriza Learning deep features for discriminative localization. Proc. Est. Acad. Sci. Biol. Ecol..

[66] van Treeck M., Cifci D., Laleh N.G., Saldanha O.L., Loeffler C.M.L., Hewitt K.J., Muti H.S., Echle A., Seibel T., Seraphin T.P. (2021). DeepMed: a unified, modular pipeline for end-to-end deep learning in computational pathology. bioRxiv.

[67] Kingma D.P., Ba J. (2014). Adam: a method for stochastic optimization. arXiv.

[68] Smith L.N., Topin N. (2017). Super-convergence: very fast training of neural networks using large learning rates. arXiv.

[69] Howard J., Gugger S. (2020). Fastai: a layered API for deep learning. Information.

[70] Howard J., Gugger S. (2020).

