# Supplementary material for: Generalizable biomarker prediction from cancer pathology slides with self-supervised deep learning: A retrospective multi-centric study
Source: Cell Rep Med. 2023 Mar 22;4(4):100980. doi: 10.1016/j.xcrm.2023.100980 (PMC10140458; doi:10.1016/j.xcrm.2023.100980)
Supplement: Document S1. Tables S1–S8 and Figures S1–S6 [file mmc1.pdf]

Cell Reports Medicine, Volume *4*

Supplemental information

Generalizable biomarker prediction from cancer pathology slides with self-supervised deep learning: A retrospective multi-centric study

Jan Moritz Niehues, Philip Quirke, Nicholas P. West, Heike I. Grabsch, Marko van Treeck, Yoni Schirris, Gregory P. Veldhuizen, Gordon G.A. Hutchins, Susan D. Richman, Sebastian Foersch, Titus J. Brinker, Junya Fukuoka, Andrey Bychkov, Wataru Uegami, Daniel Truhn, Hermann Brenner, Alexander Brobeil, Michael Hoffmeister, and Jakob Nikolas Kather

Supplementary Tables

QUASAR	TP	FN	TN	FP	DACHS	TP	FN	TN	FP
Male	110	17	808	140	Male	80	37	1042	40
Female	107	11	490	86	Female	61	32	718	29
Colon	199	25	875	178	Colon	138	59	1029	48
Rectum	7	2	391	37	Rectum	3	10	731	21
Left	177	19	861	105	Left	126	48	1230	37
Right	24	8	341	101	Right	14	21	511	32
Age	61.4	60.6	62.6	62.2	Age	70.8	69.0	68.7	66.9
Tumor Stage	2.1	2.1	2.1	2.1	Tumor Stage	2.2	2.1	2.5	2.8
BRAF^{wt}	92	18	895	140	*BRAF*^{wt}	70	53	1555	51
BRAF^{mut}	59	4	30	11	*BRAF*^{mut}	62	15	53	11
KRAS^{wt}	117	12	533	88	*KRAS*^{wt}	110	47	1073	51
KRAS^{mut}	28	8	373	56	*KRAS*^{mut}	23	18	553	16
					CIMP	95	39	1560	59
					non-CIMP	46	30	200	10

Table S1: Clinical statistics stratified by MSI biomarker test outcome for patients with CRC in the QUASAR (left) and DACHS (right) cohort at threshold value 0.5, Related to Figure 3. TP = True positive, FN = False negative, TN = True negative, FP = False positives.

QUASAR	TP	FN	TN	FP	DACHS	TP	FN	TN	FP
Male	47	18	641	204	Male	81	14	835	318
Female	41	14	357	154	Female	48	8	486	285
Colon	79	25	642	282	Colon	120	17	742	424
Rectum	5	5	309	56	Rectum	9	5	579	179
Left	74	20	650	186	Left	110	15	977	322
Right	8	10	245	145	Right	18	7	330	277
Age	66.0	63.6	62.6	61.0	Age	73.1	73.7	68.5	68.0
Tumor Stage	2.13	2.13	2.1	2.1	Tumor Stage	2.6	1.9	2.3	2.7
MSI	55	20	816	219	MSI	66	11	1135	471
non-MSI	21	8	38	72	nonMSI	55	9	50	73
KRAS^{wt}	83	28	593	176	*KRAS*^{wt}	114	21	803	357

*KRAS*mut	2	3	381	167	*KRAS*mut	8	21	426	198
					CIMP	82	16	1173	491
					non-CIMP	45	6	139	104

Table S2: Clinical statistics stratified by *BRAF* biomarker test outcome for patients with CRC in the QUASAR (left) and DACHS (right) cohort at threshold value 0.5, Related to Figure 3. TP = True positive, FN = False negative, TN = True negative, FP = False positives.

	INPT	Wang+attMIL	Ciga+attMIL	multi-input	clinical data only
**INPT**	1	0.0284	0.9526	0.0218	0.0003
**Wang+attMIL**	0.0284	1	0.0132	0.7316	<0.0001
**Ciga+attMIL**	0.9526	0.0132	1	0.0103	0.0001
**multi-input**	0.0218	0.7316	0.0103	1	<0.0001
**clinical data only**	0.0003	<0.0001	0.0001	<0.0001	1

Table S3: p-values from ANOVA analysis comparing all possible AUROC pairs of MSI models internal validation performances on Macenko normalized tiles in QUASAR, Related to STAR Methods.

	INPT	Wang+attMIL	Ciga+attMIL	multi-input	clinical data only
**INPT**	1	0.0069	0.9005	0.0221	0.4404
**Wang+attMIL**	0.0069	1	0.0346	0.8161	0.1679
**Ciga+attMIL**	0.9005	0.0346	1	0.0484	0.4796
**multi-input**	0.0221	0.8161	0.0484	1	0.1747
**clinical data only**	0.4404	0.1679	0.4796	0.1747	1

Table S4: p-values from ANOVA analysis comparing all possible AUROC pairs of *BRAF* models internal validation performances on Macenko normalized tiles in QUASAR, Related to STAR Methods.

	INPT	Wang+attMIL	Ciga+attMIL	multi-input	clinical data only
**INPT**	1	0.0002	<0.0001	0.0002	0.0001
**Wang+attMIL**	0.0002	1	<0.0001	0.7009	<0.0001
**Ciga+attMIL**	<0.0001	<0.0001	1	<0.0001	0.0001
**multi-input**	0.0002	0.7009	<0.0001	1	<0.0001

**clinical data only**	0.0001	<0.0001	0.0001	<0.0001	1

Table S5: p-values from ANOVA analysis comparing all possible AUROC pairs of MSI models validation performances on Macenko normalized tiles in DACHS, Related to STAR Methods.

	**INPT**	**Wang+attMIL**	**Ciga+attMIL**	**multi-input**	**clinical data only**
**INPT**	1	0.0001	0.0039	<0.0001	0.8309
**Wang+attMIL**	0.0001	1	<0.0001	0.0316	<0.0001
**Ciga+attMIL**	0.0039	<0.0001	1	<0.0001	0.0014
**multi-input**	<0.0001	0.0316	<0.0001	1	<0.0001
**clinical data only**	0.8309	<0.0001	0.0014	<0.0001	1

Table S6: p-values from ANOVA analysis comparing all possible AUROC pairs of MSI models validation performances on non-normalized tiles in DACHS, Related to STAR Methods.

	**INPT**	**Wang+attMIL**	**Ciga+attMIL**	**multi-input**	**clinical data only**
**INPT**	1	0.0019	0.0021	<0.0001	0.8074
**Wang+attMIL**	0.0019	1	<0.0001	0.0003	0.031
**Ciga+attMIL**	0.0021	<0.0001	1	<0.0001	0.0064
**multi-input**	<0.0001	0.0003	<0.0001	1	0.0006
**clinical data only**	0.8074	0.031	0.0064	0.0006	1

Table S7: p-values from ANOVA analysis comparing all possible AUROC pairs of *BRAF* models validation performances on Macenko normalized tiles in DACHS, Related to STAR Methods.

	**INPT**	**Wang+attMIL**	**Ciga+attMIL**	**multi-input**	**clinical data only**
**INPT**	1	0.0795	0.0177	<0.0001	0.0804
**Wang+attMIL**	0.0795	1	0.0038	0.0047	0.977
**Ciga+attMIL**	0.0177	0.0038	1	0.0002	0.0038
**multi-input**	<0.0001	0.0047	0.0002	1	0.0041
**clinical data only**	0.0804	0.977	0.0038	0.0041	1

Table S8: p-values from ANOVA analysis comparing all possible AUROC pairs of *BRAF* models validation performances on non-normalized tiles in DACHS, Related to STAR Methods.

Supplementary Figures

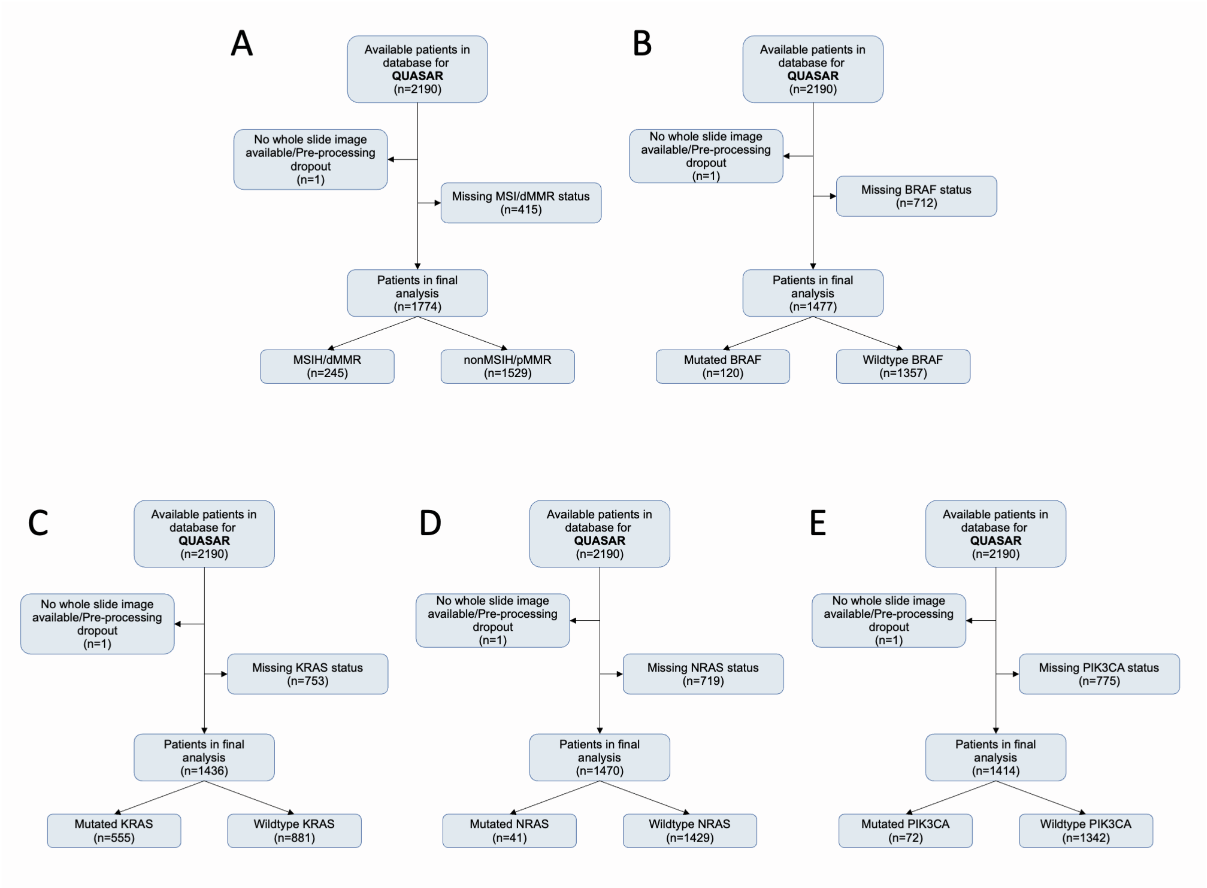

Figure S1: CONSORT charts for QUASAR, Related to Table 2 and STAR Methods. (A) MSI status, **(B)** *BRAF* status, **(D)** *KRAS* status, **(D)** *NRAS* status**, (E)** *PIK3CA* status.

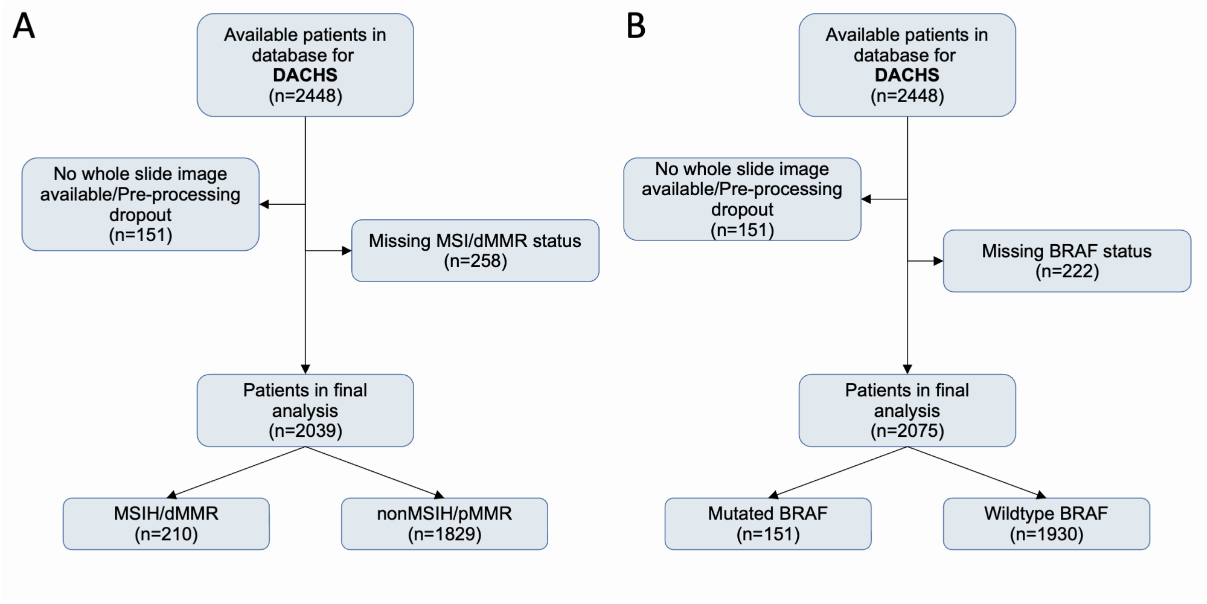

Figure S2: CONSORT charts for DACHS, Related to Table 2 and STAR methods. (A) MSI status, **(B)** *BRAF* status.

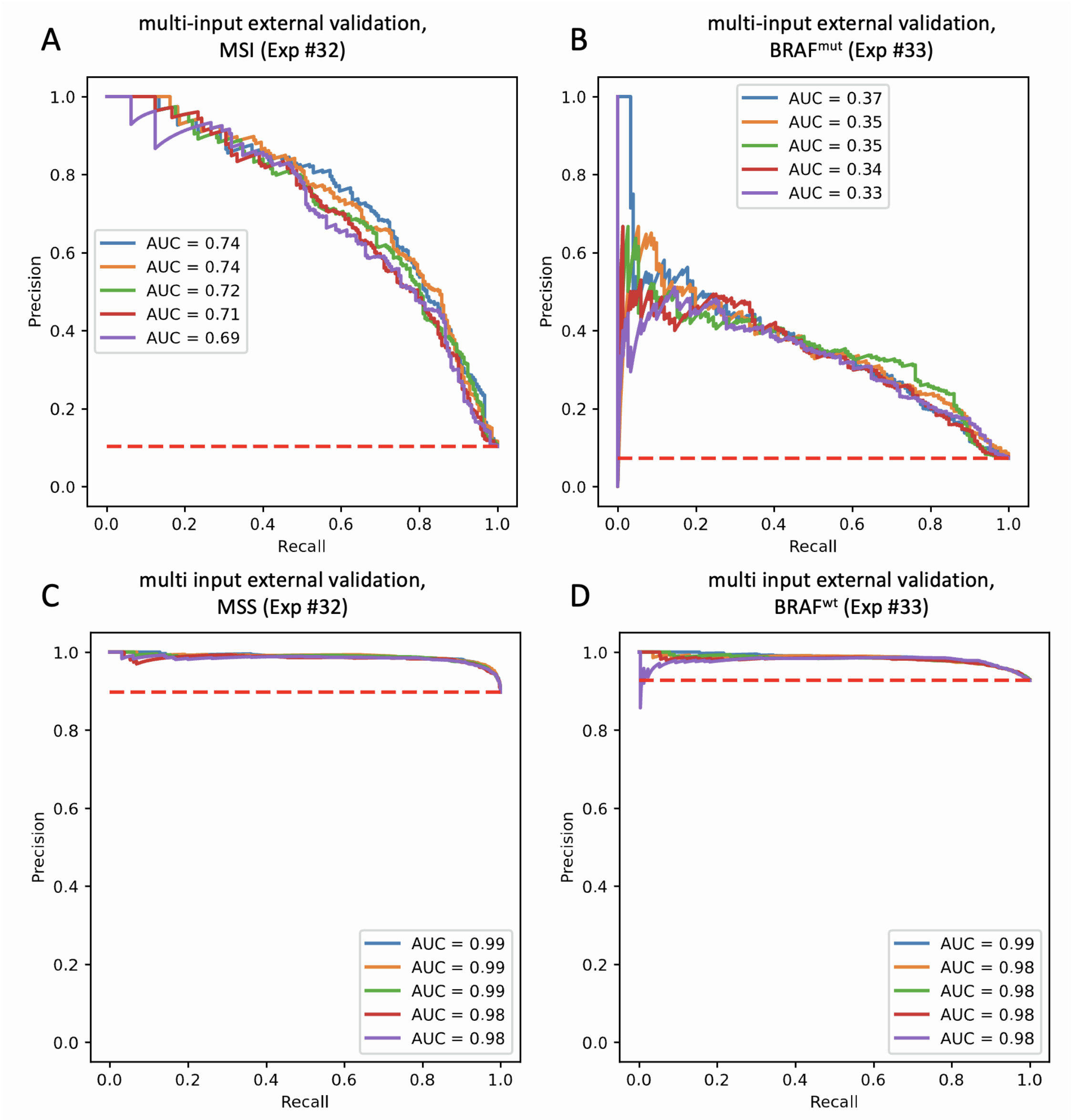

Figure S3: Precision recall curves (PRC) for the external validation (on QUASAR) of the best model (Wang-attMIL), Related to Table 1. (A) PRC for MSI detection, MSI class. **(B)** PRC for *BRAF* mutation prediction, *BRAF* mutant class, **(C)** PRC for MSI detection, non-MSI (MSS) class, **(D)** PRC for *BRAF* mutation prediction, *BRAF* wild type class. The y-values of the horizontal dotted red lines in A-D denote the fraction of true positives in the data sets and represent the precisions achieved if every patient was classified as positive.

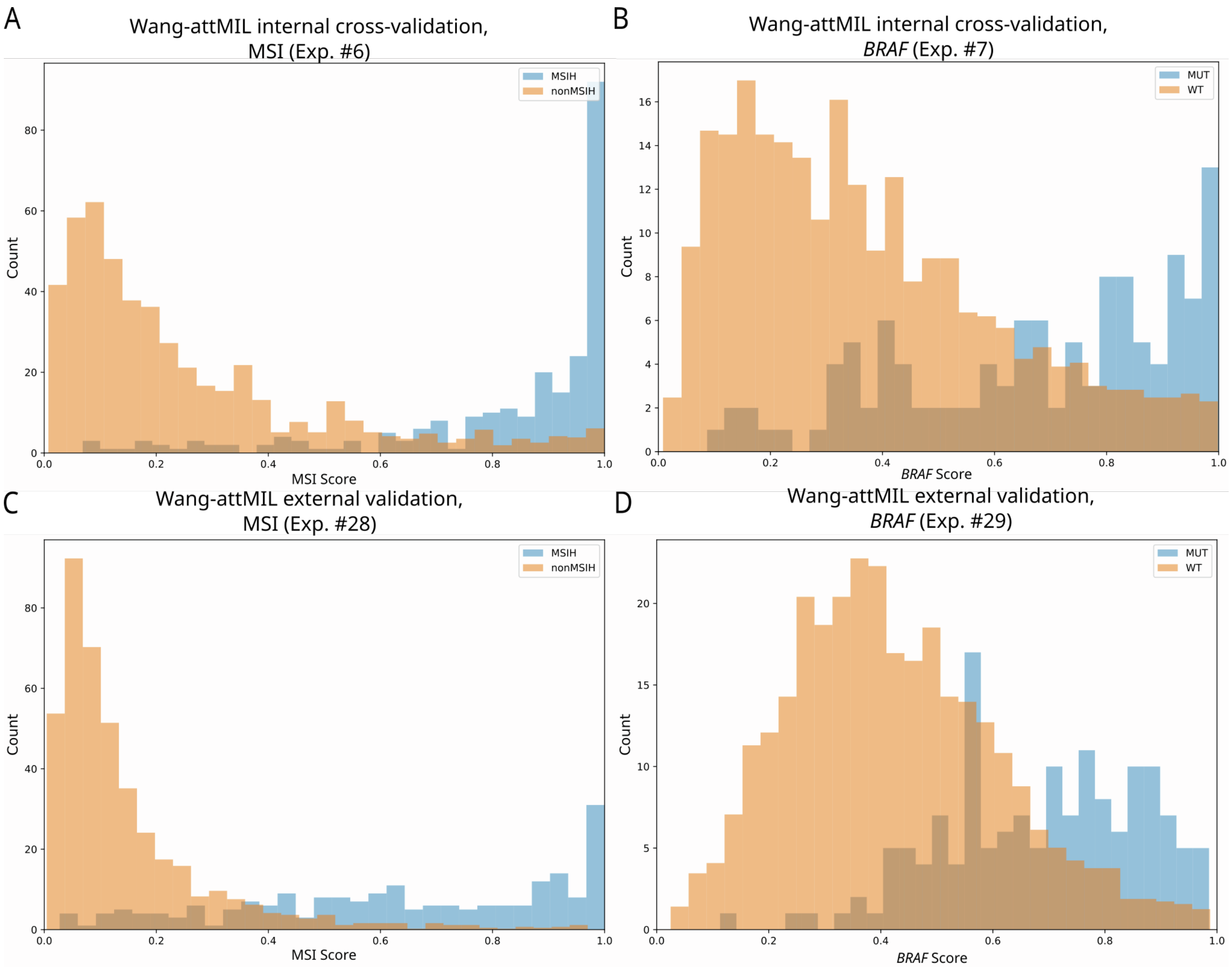

Figure S4: Quantification of the domain shift between internal and external prediction performance for the best image-only models (Wang-attMIL), Related to Table 1. (A) Distribution of model prediction scores for all patients in QUASAR, split by MSI status (ground truth), obtained via cross-validation; scores MSIH: median(m)=0.92, lower quartile(q1)=0.78, upper quartile(q3)=0.98; scores nonMSIH: m=0.169, q1=0.085, q3=0.34. **(B)** Distribution of model prediction scores for all patients in QUASAR, split by *BRAF* status (ground truth), obtained via cross-validation; scores MUT: m=0.74, q1=0.48, q3 =0.89; scores WT: median=0.32, q1=0.18, q3=0.51. **(C)** Average MSI score distribution of predictions across all models for patients in DACHS, split by MSI status (ground truth); scores MSIH: m=0.64, q1= 0.43, q3=0.90; scores nonMSIH: m=0.097, q1=0.055, q3=0.18. **(D)** Average *BRAF* score distribution of predictions across all models for patients in DACHS, split by *BRAF* status (ground truth); scores MUT: m=0.79, q1=0.66, q3=0.88; WT: m=0.37, q1=0.28, q3=0.54. Displayed frequency distributions of the more frequent class (i.e. the negative class) are rescaled to twice the frequency of the less frequent class. For internal validation, summed distributions over all five test sets, and for external validation, averaged distributions over all five models are shown.

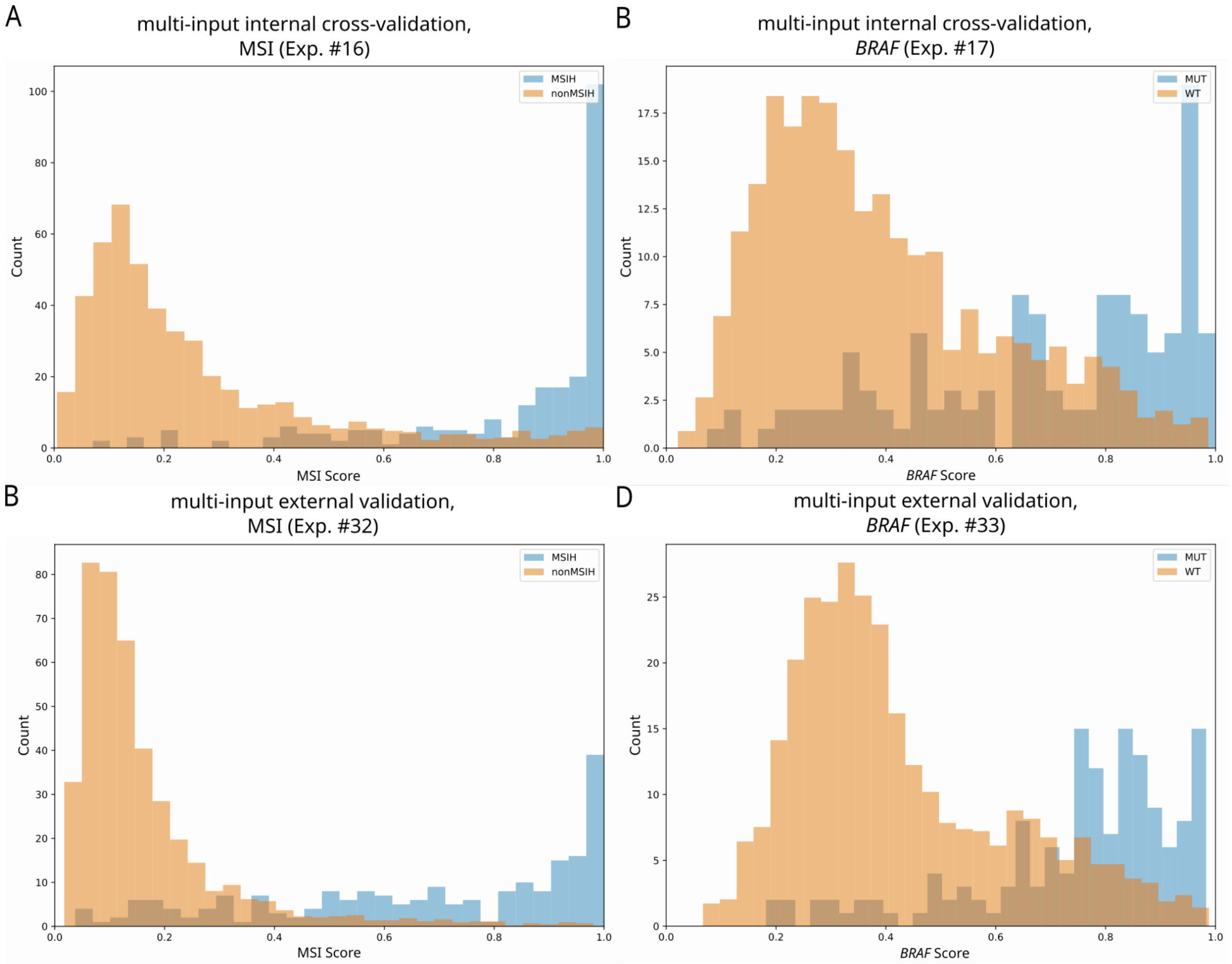

Figure S5: Prediction score distribution in internal and external sets for the best model (multi-input model), Related to Taable 1. (A) Distribution of model prediction scores for all patients in QUASAR, split by MSI status (ground truth), obtained via cross-validation; scores MSIH: median(m)=0.94, lower quartile(q1)=0.74, upper quartile(q3)=0.99; scores nonMSIH: m=0.18,q1=0.11, q3=0.32. **(B)** Distribution of model prediction scores for all patients in QUASAR, split by *BRAF* status (ground truth), obtained via cross-validation; scores MUT: m=0.78, q1=0.49,q3=0.91; scores WT: m=0.34, q1=0.23, q3=0.51. **(C)** Distribution of model prediction scores for all patients in DACHS, split by MSI status (ground truth), obtained via cross-validation; scores MSIH: m=0.72, q1=0.48, q3=0.94; scores nonMSIH: m=0.12, q1=0.08, q3=0.19. **(D)** Distribution of model prediction scores for all patients in DACHS, split by *BRAF* status (ground truth), obtained via cross-validation; scores MUT: m=0.79, q1=0.66,q3=0.88; scores WT: m=0.37, q1=0.28, q3=0.54.

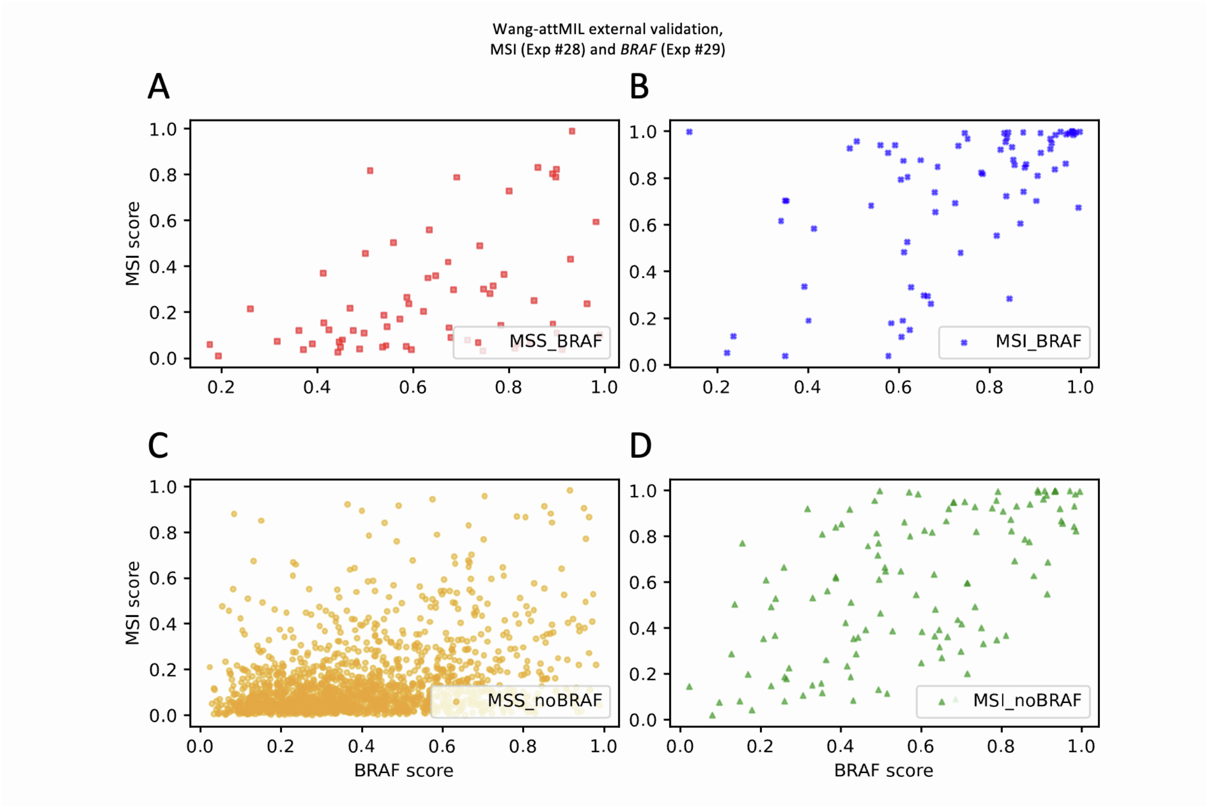

Figure S6: Correlation of prediction scores for MSI and *BRAF* status for the best image-only model (attMIL with Wang features), Related to Figure 5. (A) Correlation of MSI (vertical axis) and *BRAF* (horizontal axis) prediction score for patients with ground truth status: MSS, BRAF mut. **(B)** Correlation of MSI (vertical axis) and *BRAF* (horizontal axis) prediction score for patients with ground truth status: MSI, *BRAF* mut. **(C)** Correlation of MSI (vertical axis) and *BRAF* (horizontal axis) prediction score for patients with ground truth status: MSS, *BRAF* wild type. **(D)** Correlation of MSI (vertical axis) and *BRAF* (horizontal axis) prediction score for patients with ground truth status: MSI, *BRAF* wild type.
